# COSMIN systematic review of stigma measurement in smoking, COPD, and lung cancer: content analysis, language audit, and psychometrics

**DOI:** 10.1093/abm/kaag026

**Published:** 2026-06-11

**Authors:** Sohail Ahmad, Coral Gartner, Bernadette Richards, Henry Marshall, Julia Yamazaki-Tan, Kiernan Thompson, Kylie Morphett

**Affiliations:** NHMRC Centre of Research Excellence on Achieving the Tobacco Endgame, The University of Queensland, Herston, Queensland 4006, Australia; School of Public Health, Faculty of Health, Medicine and Behavioural Sciences, The University of Queensland, Herston, Queensland 4006, Australia; NHMRC Centre of Research Excellence on Achieving the Tobacco Endgame, The University of Queensland, Herston, Queensland 4006, Australia; School of Public Health, Faculty of Health, Medicine and Behavioural Sciences, The University of Queensland, Herston, Queensland 4006, Australia; Medical School, Faculty of Health, Medicine and Behavioural Sciences, The University of Queensland, Herston, Queensland 4006, Australia; NHMRC Centre of Research Excellence on Achieving the Tobacco Endgame, The University of Queensland, Herston, Queensland 4006, Australia; Department of Thoracic Medicine, The University of Queensland Thoracic Research Centre, The Prince Charles Hospital, Chermside, Queensland 4032, Australia; Medical School, Faculty of Health, Medicine and Behavioural Sciences, The University of Queensland, Herston, Queensland 4006, Australia; NHMRC Centre of Research Excellence on Achieving the Tobacco Endgame, The University of Queensland, Herston, Queensland 4006, Australia; School of Public Health, Faculty of Health, Medicine and Behavioural Sciences, The University of Queensland, Herston, Queensland 4006, Australia; NHMRC Centre of Research Excellence on Achieving the Tobacco Endgame, The University of Queensland, Herston, Queensland 4006, Australia; School of Public Health, Faculty of Health, Medicine and Behavioural Sciences, The University of Queensland, Herston, Queensland 4006, Australia

**Keywords:** lung cancer, chronic obstructive pulmonary disease, stigma, review, psychometrics

## Abstract

**Background:**

Stigma associated with smoking, chronic obstructive pulmonary disease (COPD) and lung cancer is prevalent and can cause distress and delayed treatment-seeking. Interventions to reduce stigma are becoming more widespread and a variety of stigma scales have been developed, but it is unknown how psychometrically sound and appropriately worded they are.

**Purpose:**

To (1) systematically identify scales that measure stigma in people who smoke, or those living with COPD or lung cancer; (2) analyze the content of items and language used; and (3) evaluate their psychometric properties.

**Methods:**

PubMed, Embase, Web of Science, and Scopus were searched to identify quantitative scales measuring stigma in smoking, COPD, or lung cancer. The Lung Cancer Stigma Communications Assessment Tool was used to assess whether the scales contained language that could exacerbate stigma amongst participants. The COSMIN tool was used to evaluate the psychometric properties of the scales.

**Results:**

We identified 962 articles for screening, of which 28 eligible scales were included. The majority (*n = *17) measured lung cancer stigma. The content of items largely aligned with theoretical dimensions of stigma. No scales were free of language that could exacerbate stigma. In relation to psychometrics, the majority of the stigma scales assessed internal consistency (*n = *25), construct validity (*n = *20), and structural validity (*n = *15). All scales were categorized as Class B, meaning that they have potential for use but require further research and psychometric testing.

**Conclusion:**

A comprehensively validated stigma scale is needed that uses non-stigmatizing, bias-free, and person-first language.

## Introduction

Chronic obstructive pulmonary disease (COPD) and lung cancer are serious public health concerns worldwide, both characterized by high incidence and mortality rates.^1^ COPD affects over 400 million people globally and is the third leading cause of death, while lung cancer is the most frequently diagnosed cancer, with 2.4 million new cases reported in 2022, and remains the leading cause of cancer-related mortality.^2–4^ In addition to the epidemiological burden, both COPD and lung cancer are among the most psychologically taxing respiratory conditions and are frequently associated with health-related stigma stemming from their diagnosis.^5^^,^^6^

Health-related stigma is a multifaceted process of devaluation applied to a person by themselves or others due to a specific health condition.^7^^,^^8^ In the context of smoking, stigma refers to negative perceptions and stereotypes of people who smoke.^9^^,^^10^ The denormalization of smoking has been effective in reducing uptake of smoking and increasing quitting.^9^ However, an unintended consequence of some anti-smoking campaigns can be stigmatization of people who smoke, and the perception that they are “inconsiderate, dirty and weak-willed.”^11–13^ People who are diagnosed with smoking-related diseases such as COPD and lung cancer frequently experience stigma.^14^ The main catalyst that perpetuates COPD- or lung cancer-associated stigma is the established link to smoking, and the attribution of personal responsibility or blame for smoking behavior.^15^^,^^16^ Media portrayals of people with smoking-related diseases often feature individuals expressing regret and self-blame about having smoked.^9^ Such depictions focus on individual responsibility and rarely discuss the role of tobacco companies in aggressively marketing these products, thereby perpetuating stigmatizing attitudes toward people who smoke and/or develop chronic respiratory disease.^17^

Stigma manifests in multiple dimensions—self (internalized or perceived) and public stigma, and exists on multiple levels such as intrapersonal, interpersonal, organizational, community-wide, or public policy level.^18–20^ Self-stigma occurs when people with COPD or lung cancer devalue themselves and internalize the idea that they are to blame for their health condition and judge themselves as unworthy of empathy.^21^ Similarly, the perception of smoking-related diseases as self-inflicted contributes significantly to public stigma.^6^ The stigma of COPD and lung cancer is also experienced by people with these diseases who have never smoked.^22^ These experiences may occur at the intrapersonal level, where individuals may experience feelings of shame or guilt related to developing these diseases^6^^,^^23^; at the interpersonal level, which involves judgmental comments from others or social isolation of those diagnosed with COPD or lung cancer^17^^,^^24–26^; and at the structural level, encompassing public portrayals of people who smoke or are living with lung disease in the media,^9^^,^^27^ as well as inequalities in funding or resources allocated to lung cancer compared with other cancers.^20^^,^^28^

Evidence from cross-sectional and longitudinal studies suggest that higher stigma in COPD and lung cancer is associated with several clinically relevant outcomes, including delays in diagnosis and treatment, reduced quality of life, and amplification of psychosocial morbidity such as anxiety and depression.^29–31^ In addition, the stigma related to smoking and smoking-related diseases disproportionately affects certain populations, including people with mental health conditions, who experience higher smoking prevalence and greater stigma.^32^ Considering these potential adverse effects of stigma, its burden should neither be underestimated nor ignored.^19^

Stigmatizing language is normalized in medicine, and healthcare providers are often unaware of its impact.^33^^,^^34^ It can exacerbate the feelings of blame, shame, and self-guilt leading to compromised psychosocial well-being of people who smoke or people who are living with COPD and lung cancer.^21^^,^  ^35^ For example, Carter-Bawa et al.^36^ revealed that existing public information often contains terms that may be perceived as stigmatizing by patients living with lung cancer. To address this, the American Cancer Society National Lung Cancer Roundtable developed the Lung Cancer Stigma Communications Assessment Tool (LCS-CAT) and encourages the use of this tool when developing any communications for lung cancer patients. LCS-CAT can serve as a helpful tool for identifying and mitigating elements of stigma in public-facing lung cancer communications.^37^

An increasing number of studies measure stigma associated with smoking and smoking-related diseases.^18^ It is currently unknown whether these questionnaires or scales contain stigmatizing language and are psychometrically robust. This is important to assess because researchers have an ethical responsibility to ensure the psychosocial well-being of participants during the research process,^36^ and there is a risk that completing a stigma questionnaire could increase feelings of stigma and self-blame. Similarly, if the measurement properties of a scale, ie, reliability, validity, and responsiveness, are not assessed, there is a risk of imprecise or biased results that might lead to wrong conclusions.^38^ Therefore, the assessment of stigma with a psychometrically robust tool is important for the emerging literature on stigma reduction interventions to enable accurate monitoring of whether such interventions to reduce stigma are effective.

To address this gap, we conducted a systematic review that provides a comprehensive overview of stigma measurement scales for administering to people who smoke or are living with COPD or lung cancer. We used LCS-CAT to assess the language compliance of existing stigma scales with International Association for the Study of Lung Cancer (IASLC) language guide, and the updated COnsensus-based Standards for the selection of health Measurement INstruments (COSMIN) guidelines to evaluate the psychometric properties of these scales.^39^^,^^40^

The objectives of this systematic review were to (1) identify the scales available to assess stigma in people who smoke or are living with COPD or lung cancer; (2) perform content analysis of these existing scales to assess the conceptual coverage of stigma; (3) examine the compliance of the identified scales with IASLC language guidelines; and (4) evaluate the psychometric properties of the existing scales to determine their suitability for future use.

## Methods

We conducted this systematic review in accordance with the Preferred Reporting Items for Systematic Reviews and Meta-Analyses (PRISMA) statement for systematic reviews^41^ and COSMIN guidelines.^39^

### Search strategy

The search strategy was developed by members of the research team with assistance from a university research librarian (G.V.) and piloted through preliminary searches. We searched 4 databases—MEDLINE (PubMed), CINAHL, Scopus, and Web of Science—from the date of inception of each database until September 2025.

The literature search was performed using a combination of search terms related to stigma, and smoking or COPD or lung cancer, and measurement terms (eg, questionnaire, survey). Search terms were modified for each database as shown in Table S1. Subsequent searches were also conducted using the names of each identified scale, questionnaire, or measure.

### Eligibility criteria and study selection

Studies were eligible if they reported on the development and/or validation of stigma scales. Questionnaires that were labeled as psychosocial scales, but that measured patients’ experiences related to stigma, were also included. Only peer-reviewed articles published in English were included. Systematic, scoping, umbrella, and narrative reviews were excluded, but their reference lists were screened to identify further relevant studies for inclusion. Studies that only applied an existing scale (parent scale) without contributing to new psychometric evidence were excluded. Furthermore, any study that did not assess stigma among people who smoke or those living with COPD or lung cancer with a scale or sub-scale, or with fewer than 3 items, was excluded. All studies that were aimed at measuring stigma-related knowledge, attitudes or practices among healthcare providers (HCPs) were excluded. Lastly, we excluded studies in which stigma sub-scales emerged through factor analysis rather than items being intentionally developed to assess stigma because these sub-scales were not originally developed to assess stigma, so their content validity was uncertain.

### Data extraction and analysis

Scales developed or used in the included studies were the items of analysis. If the scales were not fully accessible, corresponding authors were contacted to request a copy. Two authors (S.A. and J.Y.) screened the titles and abstracts separately using the key terms and inclusion and exclusion criteria. Any ambiguity was discussed and resolved by discussing with senior authors.

#### Step 1: content analysis and language audit

We extracted the items from the included stigma scales and analyzed these using a hybrid approach of deductive and inductive thematic analysis. A set of *a priori* codes was developed based on the *Health Stigma and Discrimination Framework* as proposed by Stangl et al. 2019.^42^ We used theoretical constructs, such as drivers, manifestations and outcomes of stigma, as preliminary codes. Each item was mapped to preliminary codes informed by the stigma framework.^42^ Coding was further guided by factor structures reported in 15 studies that conducted statistical factor analyses. The mapping was undertaken by a single author, and the results were subsequently presented to and reviewed by the senior authors. Concurrently, we added new codes iteratively and refined them through constant comparison. The list of identified themes, sub-themes, and examples of items was recorded.

After content analysis, we performed the language audit by adapting the LCS-CAT. LCS-CAT was developed to help content developers who are creating or modifying public-facing materials for lung cancer to identify, remove, and replace potentially stigmatizing language and imagery that could contribute to self and public stigma.^43^ It divides stigmatizing language into 3 sub-categories: labeling, blaming, and oversimplifying language. Although the primary purpose of LCS-CAT was not to perform language audits of scales, we used its definitions and examples of stigmatizing language to categorize each item as “Not Stigmatizing,” “Potentially Stigmatizing,” or “Definitely Stigmatizing.” In addition, those items that were categorized as “Potentially Stigmatizing,” and “Definitely Stigmatizing” were given a yes/no rating for labeling language (focusing on the disease/behavior and not the person), blaming (using words and phrases that show negativity and judgment), and oversimplifying language (reducing a person to single word and not reflecting the complexity of the lung condition). If the reviewer found an item statement “Potentially” or “Definitely Stigmatizing” but could not categorize it into 1 of the 3 categories of labeling, blaming, and oversimplifying language, it was categorized as “other.”

Initially, S.A. rated all items across all the scales to maintain consistency in scoring. The second ratings were conducted by the other members of the research team (K.M., H.M., B.R., J.Y.-T., and C.G.), with items equally distributed among them. Each scale, therefore, received 2 independent ratings to assess the compliance with non-stigmatizing language outlined in the LCS-CAT.^36^ Discrepancies between the ratings were discussed by this team until a consensus was reached.

#### Step 2: psychometric analysis

We used the COSMIN checklist to assess the psychometric properties of the included studies.^44^^,^^45^ COSMIN is an initiative that aims to “improve the selection of outcome measurement instruments of health outcomes by developing and encouraging the use of transparent methodology and practical tools for selecting the most suitable outcome measurement instrument in research and clinical practice.”^39^ COSMIN assesses 9 psychometric properties of scales, which can be organized into 3 main domains.

- *Validity* (content validity, structural validity, hypothesis testing for construct validity, and cross-cultural validity) assessing whether the scale measures the intended construct comprehensively and accurately.
- *Reliability* (internal consistency, reliability, and measurement error) to measure the extent to which scores are free from random and systematic error.
- *Responsiveness*, which evaluates the scale’s ability to detect meaningful change over time.

##### Step 2.1: risk of bias (RoB) assessment

We evaluated the methodological quality of each scale using the COSMIN risk of bias checklist. S.A. assessed the RoB for each included scale. A subsample (10 studies) of the COSMIN checklist appraisal data was checked with another author (K.T.). The COSMIN-RoB Checklist consists of 10 dimensions, which include scale development (22 items), content validity (42 items), structural validity (4 items), internal consistency (4 items), cross-cultural validity/measurement invariance (4 items), reliability (8 items), measurement error (6 items), criterion validity (3 items), hypothesis testing of construct validity (7 items), and responsiveness (13 items). The response options for each item are: “very good,” “adequate,” “doubtful,” “inadequate,” and “NA (not applicable).” We followed the “worst score counts” principle for the RoB assessment of all measurement properties as proposed by COSMIN guidelines. For instance, in RoB assessment for content validity, we considered the lowest quality rating among the 3 key aspects of content validity: relevance, comprehensiveness, and comprehensibility. If relevance and comprehensibility were supported by moderate quality evidence, but comprehensiveness was supported by low quality evidence, the overall content validity rating was classified as low.

##### Step 2.2: assessment of measurement properties

Two researchers (S.A. and K.T.) independently extracted the results and evaluated them by the criteria as stipulated in the COSMIN guidelines. Each measurement property was rated as “sufficient (+),” “insufficient (−),” or “indeterminate (?).” We did not evaluate the measurement property if that was rated as “NA” in RoB assessment.

##### Step 2.3: grading evidence

We applied the modified Grading of Recommendations Assessment, Development, and Evaluation (mGRADE).^23^ We rated the properties of the included stigma scales based on 4 factors: risk of bias, inconsistency, imprecision, and indirectness. Each measurement property was rated as “high,” “moderate,” “low,” and “very low.” When information on a specific measurement property was not extracted, it was recorded as “NCA (not clear if the property was assessed).”

##### Step 2.4: recommendations based on COSMIN

Lastly, we ranked all the included scales into 3 classes according to COSMIN guidelines:

- Class A: It can be recommended for use and results obtained with the scale can be trusted.
- Class B: It has the potential for recommendation but needs further research into the measurement properties.
- Class C: It should not be recommended for use.

**Figure 1:**
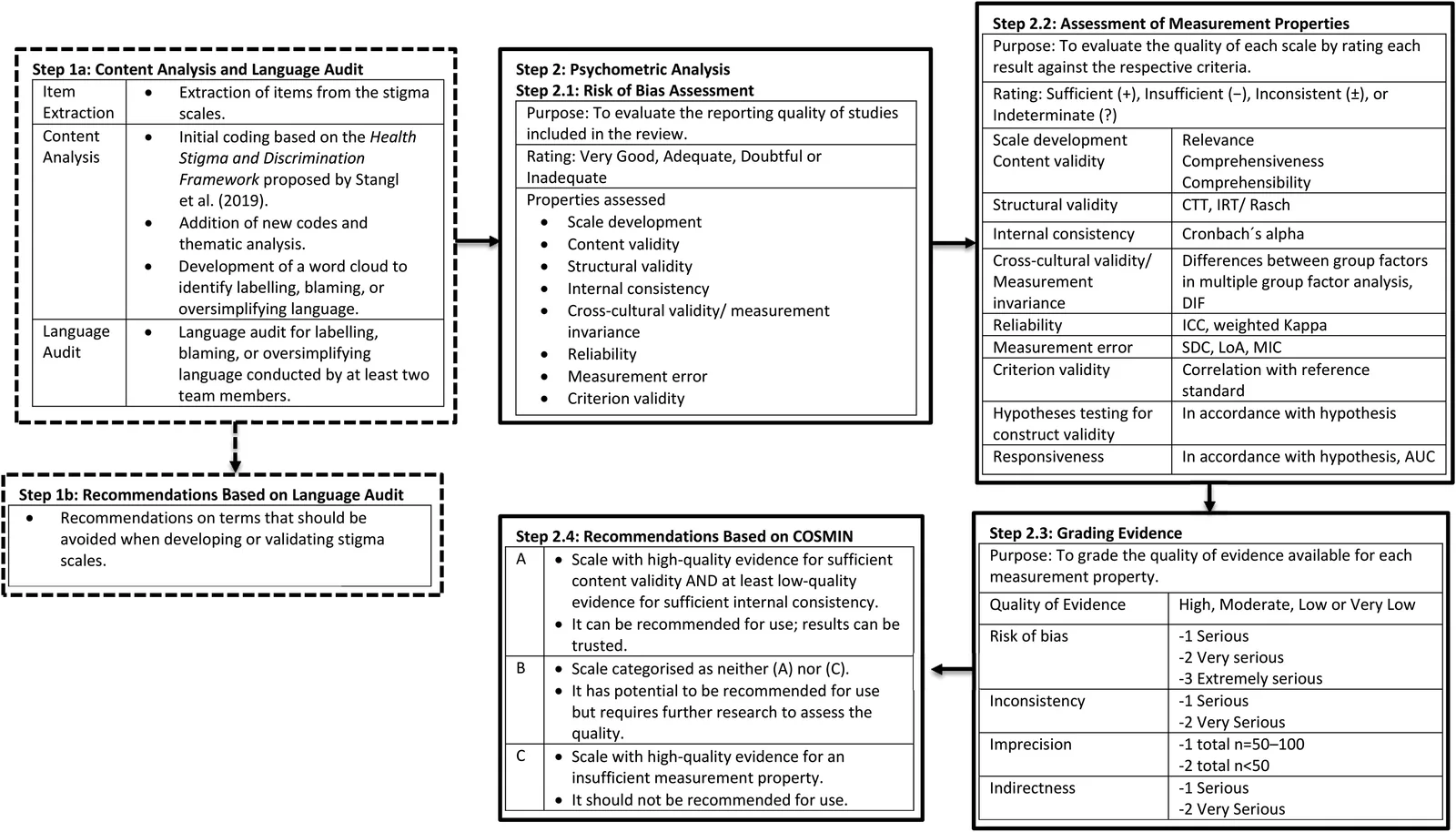
Overview of the content analysis, language audit, and COSMIN methodology.

The overview of study methodology is shown in Figure 1.

## Results

**Figure 2:**
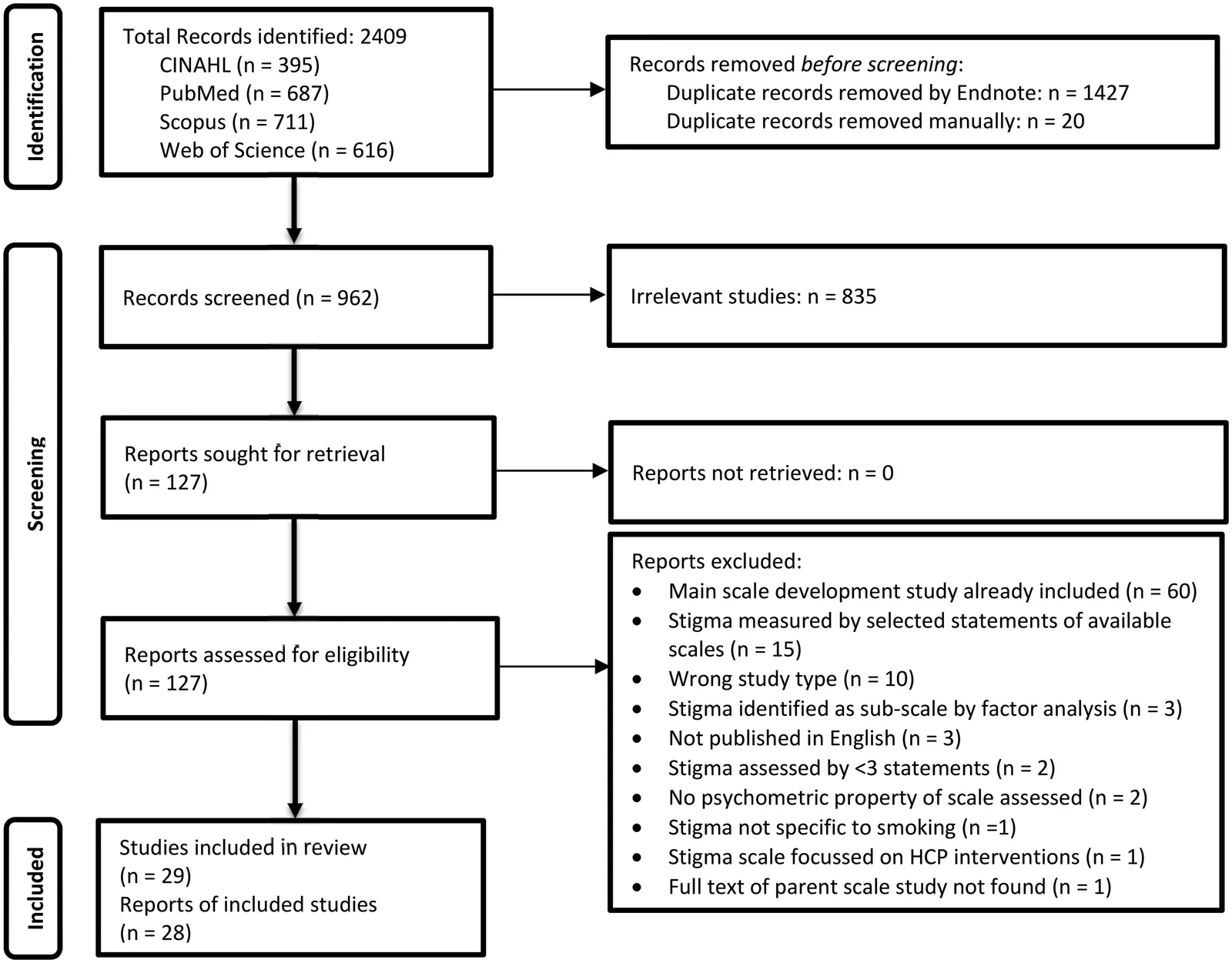
PRISMA flow diagram of articles included in this review.

The search strategy identified 2409 records. After excluding duplicates, 962 articles were screened, leading to the inclusion of 29 articles and the identification of 28 different stigma scales. For 1 scale the psychometric properties were reported in 2 different studies. The PRISMA flow diagram is shown in Figure 2.

Eligible studies were conducted in the USA (*n = *15), China (*n = *3), France (*n = *2), South Korea (*n = *2), Canada (*n = *1), Australia (*n = *1), UK (*n = *1), Mexico and Uruguay (*n = *1), Mexico (*n = *1), and Germany (*n = *1). The sample sizes of these studies ranged from 62 to 9966, with included studies measuring stigma in the following populations: females (*n = *1), people with lung cancer (*n = *12), people with COPD (*n = *2) and people who smoke (*n = *8). Some studies assessed the stigma of different types of cancers, including lung cancer (*n = *6). Further details of study characteristics are reported in Table S2. The majority of scales (*n = *20) analyzed both external and internalized stigma. The total number of items ranged from 3 to 60. The measurement characteristics of the scales are shown in Table 1.

**Table 1:** Characteristics of included stigma scales (*n = *28).

Scale (source)	Concept measured	Target population	Country	Sample size	Origin of scale	Number of items	(Sub)scale names (number of items)	Response options
**Stigma scales for lung cancer**								
**Lung cancer stigma and discrimination scale^46^**	Lung cancer stigma and discrimination	People with lung cancer receiving oncologic treatment	United States	108	Adapted from the Cancer Responsibility and Regret Scale (CRRS), HIV Stigma Scale, and an author-constructed measure of perceived subtle discrimination.	60	Internalized stigma (8) Constrained disclosure (2) Perceived subtle discrimination (50)	7-Point Likert scale (1-7) for internalized stigma and constrained disclosure 4-Point Likert scale (0: never; 4: very often) for perceived subtle discrimination
**Social impact scale^47^**	Social impact of lung cancer	People with lung cancer recruited from a tertiary cancer centre	China	283	Adapted from the original Social Impact Scale for cancer stigma.	24	Social rejection (9) Financial insecurity (3) Internalized shame (5) Social isolation (7)	4-Point Likert scale (1–4)
**Social Impact Scale^48^**	Lung cancer stigma in post-chemotherapy	People receiving chemotherapy for stage II–IV non-small cell lung cancer	United States	95	Adapted from previous studies on stigma in HIV and cancer populations.	24	Social rejection (9) Financial insecurity (3) Internalized shame (5) Social isolation (7)	4-Point Likert scale (1–4)
**Lung Cancer-Related Stigma Measure^49^**	Stigma of lung cancer	People with advanced lung cancer	United States	62	Derived from prior stigma scales, with additional lung cancer–specific items.	6	Single scale measuring overall stigma (6)	4-Point Likert scale ranging from 1 (not at all) to 4 (very much).
**Lung Cancer Stigma Inventory^50^**	Stigma of lung cancer	People with lung cancer	United States	231	Concept elicitation, item generation, and refinement informed by a literature review, individuals with lung cancer, and experts.	25	Internalized stigma (9) Perceived stigma (10) Constrained disclosure (6)	5-Point Likert scale (1–5)
**Cancer Responsibility and Regret Scale^51^**	Lung cancer responsibility and regret	People with lung cancer	United States	213	Developed based on clinical interactions with a lung cancer support group and the literature.	11	Personal responsibility (4) Regret (3) Medical stigma (4)	7-Point Likert scale (1–7)
**Lung Cancer Stigma Adaptation of the Shame and Stigma Scale^52^**	Lung cancer stigma	People diagnosed with lung cancer who had a smoking history	United States	141	Adapted from the Shame and Stigma Scale for head and neck cancers.	21	Perceived stigma (10) Internalized stigma (11)	4-Point Likert scale (0–3)
**Cataldo Lung Cancer Stigma Scale^10^**	Lung cancer stigma	People with lung cancer	United States	186	Adapted from the HIV Stigma Scale and validated in lung cancer patients.	31	Stigma and shame (11) Social isolation (10) Discrimination (5) Smoking-related stigma (5)	4-Point Likert scale (1–4)
**Short Version of the Cataldo Lung Cancer Stigma Scale^53^**	Lung cancer stigma	People with lung cancer	United States	94	Adapted from the original 31-item Cataldo Lung Cancer Stigma Scale.	21	Shame and blame (8) Social isolation (9) Discrimination (4)	4-Point Likert scale (1–4)
**Chinese Version of the Cataldo Lung Cancer Stigma Scale^54^**	Lung cancer stigma	People with lung cancer	China	150	Adapted from the original Cataldo Lung Cancer Stigma Scale.	27	Stigma and shame (14) Social isolation (6) Discrimination (3) Smoking status (4)	4-Point Likert scale (1–4)
**Shortened Version of the Cataldo Lung Cancer Stigma Scale—Chinese version^55^**	Lung cancer stigma	People with lung cancer	China	394	Adapted from the original Cataldo Lung Cancer Stigma Scale.	22	Stigma and shame (6) Social isolation (8) Discrimination (4) Smoking (4 items)	4-Point Likert scale (1–4)
**Shortened Version of the Cataldo Lung Cancer Stigma Scale—Mexican version^56^**	Lung cancer stigma	People with lung cancer	Mexico	265	Adapted from the original Cataldo Lung Cancer Stigma Scale.	17	Stigma and shame (5) Social isolation (5) Discrimination (3) Smoking (4)	4-Point Likert scale (1–4)
**Stigma Scales for Cancer**								
**Illness-Related Stigma Scale^57^**	Illness-related stigma	People with suspected or newly diagnosed cancers	United States	303	Adapted from the stigma scale developed by Fife and Weiss (2000) for HIV/AIDS and cancer.	5	Illness-related stigma subscale for internalized shame (5)	4-Point Likert scale (1–4)
**Korean Cancer Stigma Scale^58^**	Health-related stigma across cancers	Inpatients and outpatients diagnosed with cancer	South Korea	247	Adapted from the Cataldo Lung Cancer Stigma Scale.	25	Social isolation (5) Distancing/avoiding (4) Discrimination (4) Guilt (5) Attribution (3) Lack of medical support (3)	4-Point Likert scale (1–4)
**Social Impact Scale^59^**	Social impact of 4 types of cancer (including lung cancer)	People with breast, colon, lung or prostate cancer	Germany	858	Adapted from the original Social Impact Scale.	24	Social Rejection (6) Isolation (9) Financial insecurity (3) Internalized shame (6)	4-Point Likert scale (0–3)
**Cancer Stigma Scale^60^**	Public stigma towards cancer	Participants from the general population	United Kingdom	1205	Derived from stigma frameworks and adapted for cancer using existing illness stigma scales.	25	Awkwardness (5) Severity (5) Avoidance (5) Policy opposition (3) Personal responsibility (4) Financial discrimination (3)	6-Point Likert scale (1–6)
**Explanatory Model Interview Catalogue (EMIC) - Perceived Stigma Subscale^61^**	Perceived stigma in head & neck, and lung cancer	People with head and neck cancer and with lung cancer	Canada	206 (LC: 107)	Adapted from Weiss (1997) for culturally sensitive stigma measurement in physical and mental health conditions.	13	Concerns about disclosure of illness, diminished sense of identity and concerns about social rejection of self and family	4-Point Likert scale (1–4)
**Perceived Cancer-Related Stigma Scale^62^**	Perceived stigma of 3 types of cancer	People with cancer	United States	172 (LC: 96)	Derived from qualitative input from a focus group of lung cancer survivors.	6	Single dimension of perceived stigma (6)	5-Point Likert scale (1–5)
**Stigma scales for COPD**								
**COPD-related Stigma Scale^16^**	COPD-related stigma	People living with COPD	United States	148	Adapted from the 60-item COPD-related stigma scale, developed based on findings from a qualitative study on stigma in people with COPD and the HIV Stigma Scale.	24	Social stigma (12) Felt stigma (6) Anticipated stigma-oxygen (3) Smoking-related stigma (3)	4-Point Likert scale (1–4)
**Chronic Illness Anticipated Stigma Scale (CIASS), (Sub-scale)^29^**	Chronic illness anticipated stigma	People who smoke or previously smoked, with or without COPD or other chronic illnesses	Australia	556	Adapted from the Chronic Illness Anticipated Stigma Scale.	5	Sources of anticipated stigma Healthcare workers (sub-scale) (5)	5-Point Likert scale (1–5)
**Smoking-related stigma scales**								
**Smoker Self-Stigma Questionnaire^63^**	Self-stigma of smoking	Adults who smoke	United States	592	Developed from 13 validated addiction and behavioral stigma scales, adapted to smoking.	18	Enacted stigma (6) Felt stigma (6) Internalized stigma (6)	7-Point Likert scale (1-7)
**Smoking Stigma During the COVID-19 Pandemic^64^**	Stigma of smoking during COVID-19	Adults, including people who smoke and don’t smoke	South Korea	7293	Derived from existing stigma research, tailored to the COVID-19 context.	9	Social stigma (5) Personal stigma (4)	5-Point Likert scale (1: Not at all, 5: Extremely)
**Pregnant Smoker Stigma Scale—Self-Stigma^65^**	Self-stigma of smoking in pregnancy	Pregnant women who smoke	France	142	Adapted from the Public Stigma Scale (P3S-PS) for pregnant women who smoke.	51	Perceived Stigma: Derogatory cognitions (12) Negative emotions and behaviors (8) Personal distress (4) Information provision (2) Internalized Stigma: Derogatory cognitions (12) Negative emotions and behaviors (7) Personal distress (4) Information provision (2)	6-Point Likert scale (1–6)
**Pregnant Smoker Stigma Scale—Public Stigma^66^**	Public stigma of smoking in pregnancy	Adults from the French general population	France	342	Developed from qualitative data on stigma towards pregnant women who smoke, involving 100 participants recruited from social media.	26	Derogatory cognitions (12) Negative emotions and behaviors (8) Personal distress (4) Information provision (2)	6-Point Likert scale (1–6)
**Smoking-Related Stigma^67^**	Stigma of smoking	People who smoke and participated in the International Tobacco Control Policy Evaluation Survey	Mexico, Uruguay	Mexico: 6670 Uruguay: 3296	Based on Link and Phelan’s conceptual framework for stigma (2006).	3	Feeling uncomfortable (1) Negative stereotypes (1) Perceived marginalization (1)	5-Point Likert scale (1–5)
**Internalized Stigma of Smoking Inventory^11^**	Internalized stigma of smoking	People who smoke and are living with mental health diagnoses	United States	956	Adapted from the Internalized Stigma of Mental Illness.	8	Self-stigma (3) Felt stigma (3) Discrimination (2)	4-Point Likert scale (1–4)
**Smoking-Related Stigma Scale^68^**	Stigma of smoking	People who currently smoke	United States	811	Derived from mental health stigma scales, adapted to smoking.	11	Devaluation (2) Differential treatment (3) Social withdrawal (3) Secrecy (3)	4-Point Likert scale (0–3)
**Smoker-Related Stigma Scale^69^**	Stigma of smoking	Persons who currently smoke and people who previously smoked	United States	816	Adapted from mental health stigma scales, focusing on smoking contexts.	5	Social distance (2) Devaluation (2) General stigma perception (1)	4-Point Likert scale (1–4)

**Table 2:** Summary of themes and subthemes identified through content analysis.

Stangl’s Stigma framework domain	Theme	Sub-theme	Examples
**Drivers**	Attribution of Responsibility and Blame	Self-blame and responsibility	When it comes to my cancer, I am to blame. I accept personal responsibility for getting cancer.
		Explicit blame	People have said that those with lung cancer get what they deserve.
		Regret, shame, guilt	If I had done things differently, I probably would not have developed lung cancer. I have no regrets about the way I’ve lived my life. When it comes to my cancer, I have nothing to be ashamed of.
**Manifestations**	Experiences of Discrimination	Nonverbal discrimination	Since being diagnosed with lung cancer, how often do you experience any situations in which, because of your lung cancer, your: friends, partner (if applicable), family (other than partner), medical team, and acquaintances/coworkers: Avoid making eye contact with you. Avoid being physically close to you. Show discomfort toward you.
		Verbal/attitudinal discrimination	People discriminate against me because I am a smoker. People often treat me disrespectfully just because I am a smoker.
		Social distancing	I stopped socializing with some because of their reaction. For a smoker, it is awkward to socialize with non-smokers.
		Systemic and institutional discrimination	It is acceptable for banks to refuse to make loans. It is acceptable for insurance companies to reconsider a policy if someone has cancer.
**Stigma “Marking”**	Societal Labeling and Rejection	Social rejection	Most people with COPD are rejected when others find out. Some family members have rejected me because of my illness.
		Self-exclusion	It is easier to avoid new friendships than worry about telling someone.
		Societal labeling	Most people believe that a person who has COPD is unclean. Smoking is what weak-willed people do.
**Manifestations**	Constrained Disclosure	Self-concealment	I hide my lung cancer diagnosis from others. I have had an urge to keep my lung cancer a secret.
		Controlled disclosure	I have told people close to me to keep the fact that I have COPD a secret.
**Manifestations**	Healthcare-Associated Stigma	Healthcare providers’ blame and discrimination	Nurses who have cared for me seem to blame me for my cancer. My doctor acts as if I am to blame.
		Mistrust of medical care	Doctors have taken steps that have made my cancer worse. I feel that I have gotten worse medical care than other patients with cancer.
**Outcomes**	Consequences of Stigma and Coping Strategies	Loneliness and isolation	I feel lonely more often than usual. I have lost friends by telling them I have lung cancer.
		Emotional consequences	Having lung cancer makes me feel like I’m a bad person.
		Employment consequences	People with lung cancer lost jobs when employers learn.
		Self-Compassion	There is nothing I could have done to keep myself from getting cancer.

### Content analysis and language compliance audit

Our content analysis of scale items identified 6 themes: attribution of responsibility and blame, experiences of discrimination, societal labeling and rejection, constrained disclosure, healthcare-associated stigma, and consequences of stigma and coping strategies. Seventeen stigma scales are individual-centric, measuring internalized and/or perceived stigma, and 2 scales measured public stigma. We found that certain components of Stangl’s stigma framework are not addressed in the existing stigma scales, these included the facilitators that mitigate stigma (responsible media narratives), intersectional stigma (gendered stigma), secondary stigma (caregiver/family stigma), institutional discrimination (healthcare bias), and meso-level (organizational) and macro-level (policy and social) determinants of stigma.^42^ The details of identified themes and sub-themes and examples are shown in Table 2.

**Figure 3:**
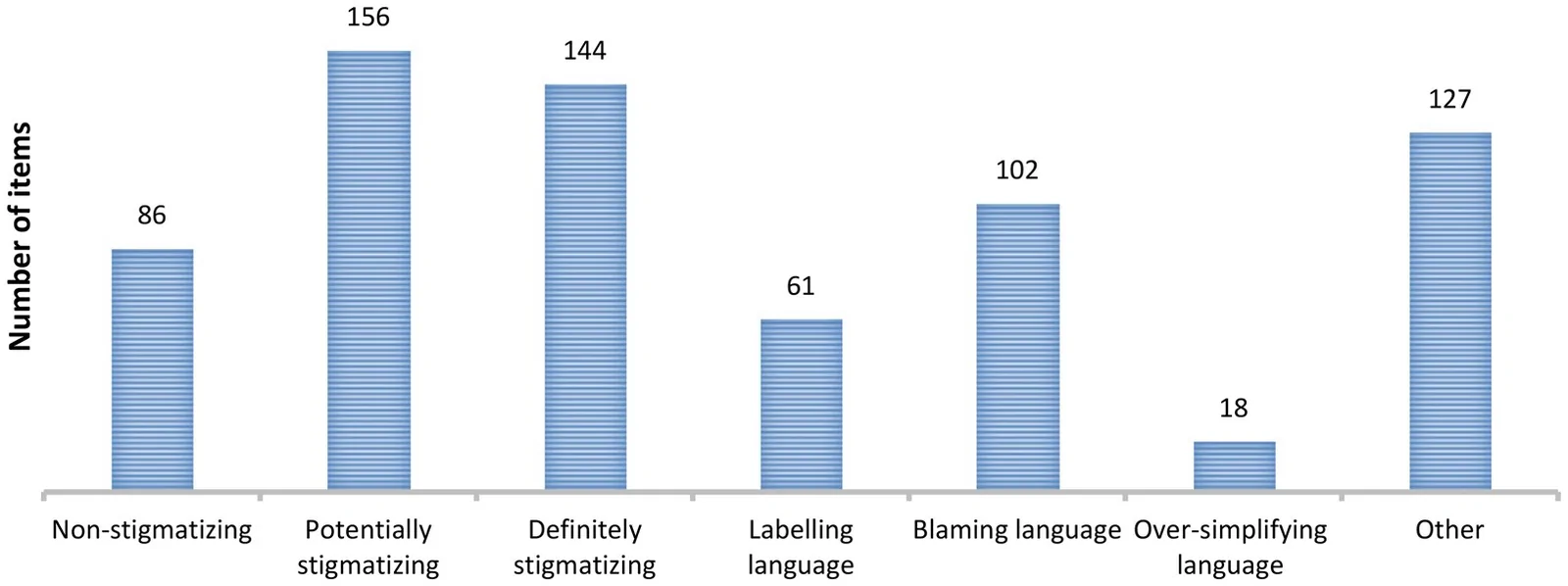
Key results of stigma language audit.

**Figure 4:**
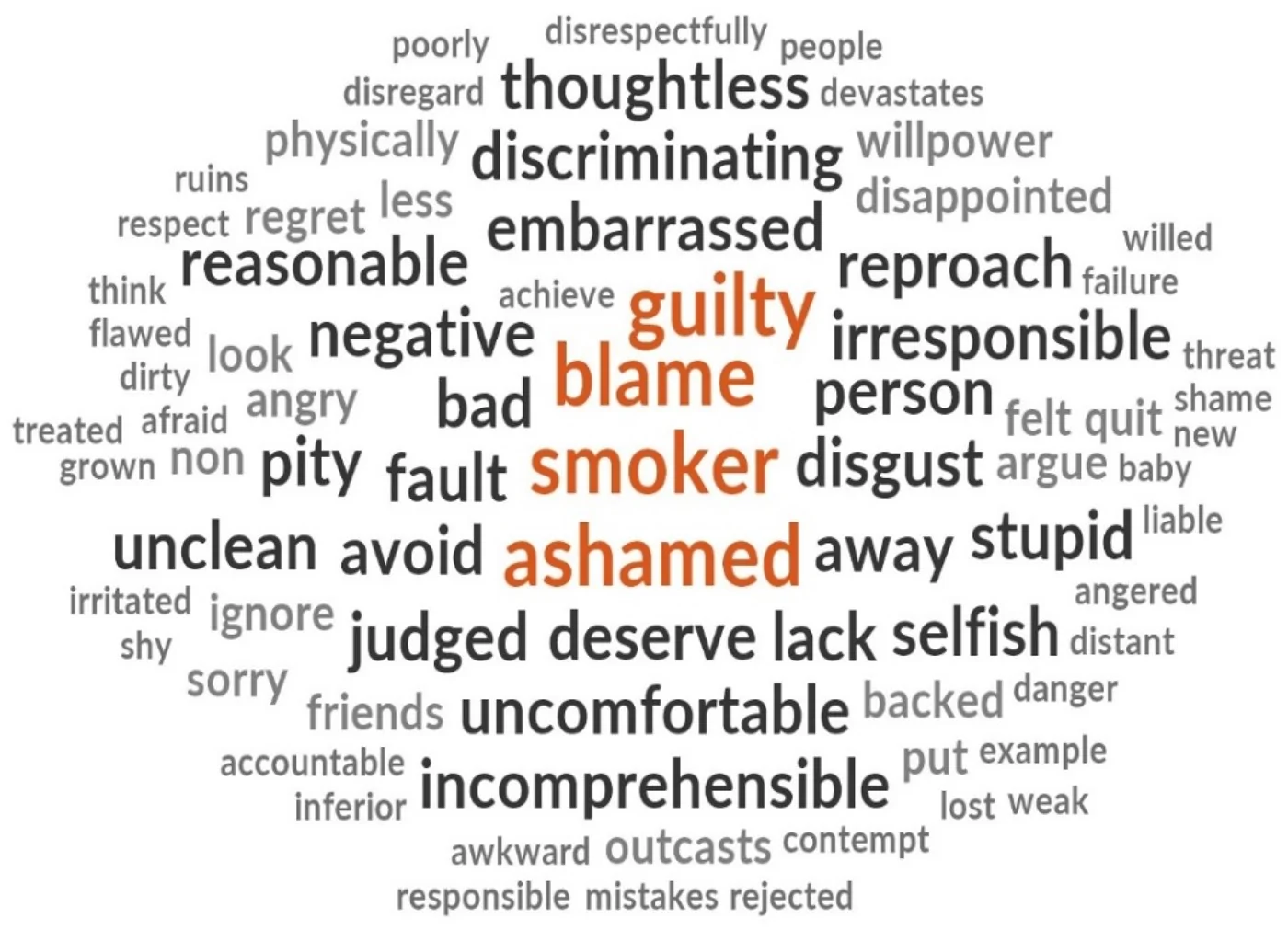
Word cloud of stigmatizing language generated in NVivo.

In the language audit, we extracted 386 statements from the included scales and found no scales that were free of labeling, blaming, or oversimplifying language. The proportion of items in scales that were identified as potentially or definitely stigmatizing ranged from 24% to 100%. The results of the language audit are shown in Figure 3. In addition, we prepared a word cloud based on these terms (Figure 4) to gauge which terms were most commonly used. The larger words represent their greater use across items.

### Methodological quality assessment and rating of psychometric properties

Of the 28 scales that met the eligibility criteria, 12 studies assessed content validity, 15 assessed structural validity, 25 assessed internal consistency, 2 assessed cross-cultural validity, 7 assessed reliability by test-retest approach, 4 assessed criterion validity and 20 used hypotheses testing for construct validity (convergent and know-group validity). None of the studies examined the measurement error or responsiveness of the included scales.

#### Development of stigma scales

All scales were developed with a clear theoretical foundation, informed by a literature review of the construct of interest or the adaptation of existing scales (primarily from HIV and mental health research). COSMIN guidelines suggest involving consumers in concept elicitation phase, and pilot testing. Sixteen studies reported that they involved experts or consumers as part of the conceptualization and pilot testing of the scale. Per-study details of the concept measured, intended context of use, item development processes, and consumer involvement in scale development are presented in Table S3. Ratings of methodological quality, measurement properties, and certainty of evidence for all included studies are reported in Table S4.

#### Content validity

Twelve studies reported on content validity.^10^^,^^16^^,^^32^^,^^50^^,^^54^^,^^58^^,^^62^^,^^63^^,^^65^^,^^66^^,^^68^^,^^69^ Only 2 studies followed the best practice proposed by COSMIN, ie, conducted a concept elicitation study (the qualitative phase in scale development) and assessed content validity by involving both experts and target participants. These studies received a sufficient “+” rating for content validity with moderate-quality evidence, downgraded due to risk of bias (RoB).^16^^,^^65^ Two additional studies also received a sufficient “+” rating with moderate-quality evidence.^10^^,^^66^ The remaining 8 studies were rated sufficient “+” for content validity, but the quality of evidence was either low or very low, primarily due to the lack of patient/consumer involvement in the content validity or pilot studies, and concerns related to RoB, including lack of information on interviewer training, use of an appropriate interview guide, verbatim transcription, independent coding, and evidence of data saturation.

#### Structural validity

Fifteen studies assessed structural validity using either exploratory factor analysis (*n = *10),^10^^,^^19^^,^^50^^,^^51^^,^^53^^,^^54^^,^^55^^,^^58^^,^^66^^,^^69^ or confirmatory factor analysis with a pre-specified model (*n = *5).^32^^,^^56^^,^^60^^,^^63^^,^^65^ Of these, 9 studies received a “+” rating for structural validity and were supported by high-quality evidence. The remaining 6 studies did not meet the COSMIN construct validity requirements and were rated “–,” with the quality of evidence ranging from moderate to very low. All 10 studies that used exploratory factor analysis were rated “A, Adequate” for methodological quality.

#### Internal consistency

Twenty-five studies reported on internal consistency.^10^^,^^16^^,^^32^^,^^46^^,^^47^^,^^48^^,^^50^^,^^51^^,^^52^^,^^53^^,^^54^^,^^55^^,^^56^^,^^57^^,^^58^^,^^59^^,^^60^^,^^61^^,^^62^^,^^63^^,^^64^^,^^65^^,^^66^^,^^68^ All these studies calculated Cronbach’s α, and their methodological quality was rated as “VG” (very good). Seventeen studies met the COSMIN requirements for internal consistency and received a “+” rating, whereas 6 studies failed to meet the COSMIN criteria (ie, Cronbach’s α < .70) and were rated “–.” The remaining 2 studies received a “?” rating due to insufficient evidence for unidimensionality^62^ and failure to report Cronbach’s α values for subscales.^47^

#### Cross‑cultural validity/measurement invariance

Of the studies included in this systematic review, only 2 conducted cross-cultural validity testing.^63^^,^^66^ Both were rated high in methodological quality and received a “+” rating for measurement properties.

#### Reliability

Seven studies assessed reliability.^50^^,^^53^^,^^54^^,^^56^^,^^60^^,^^65^^,^^66^ All were rated positively and were supported by high-quality evidence, due to sufficient details regarding the stability of constructs during the retest period and consistency of testing conditions.

#### Criterion validity

Criterion validity was assessed in 4 studies.^10^^,^^16^^,^^50^^,^^54^ All reported correlations were ≥0.70 with the reference standard, earning a “VG” rating for methodological quality and a “+” rating for measurement properties. The quality of evidence for all 4 studies was high.

#### Hypotheses testing for construct validity

Twenty studies assessed construct validity through hypotheses testing, including convergent validity and known-groups validity (KGV).^10^^,^^16^^,^^29^^,^^32^^,^^46^^,^^47^^,^^48^^,^^49^^,^^51^^,^^52^^,^^55^^,^^58^^,^^60^^,^^61^^,^^62^^,^^63^^,^^65^^,^^66^^,^^67^^,^^70^ Among these, 3 studies assessed both convergent and KGV, 12 assessed KGV alone, and 5 assessed only convergent validity. All these studies were rated “+” and supported by high-quality evidence, except for 3 studies, for which the quality of evidence ranged from moderate to low due to small sample sizes^48^^,^^49^ and indirectness.^58^

#### Grading evidence and recommendations for the included scales

We graded the evidence for each reported measurement property of each scale based on 4 parameters: risk of bias, inconsistency, imprecision, and indirectness. No scales were categorized as Class A or Class C. Based on the COSMIN guidelines, we assigned a Class B recommendation to all included scales, suggesting that they have potential for use but require further research to confirm the quality of scale. A summary of findings with the evidence for each measurement property of each scale is shown in Table 3.

**Table 3:** Summary of findings with the evidence for each measurement property of each scale.

Scale	CV			SV	IC	MI/CCV	REL	ME	CrV	CoV		RESP	CL
	REL	COMP	COMB							CON	KGV	Pre-Post	
**LCSDS^46^**					+/High					+/High			B
**SIS^47^**					?					+/High	+/High		B
**SIS^48^**					+/Moderate					+/Moderate			B
**LCRSM^49^**											+/Low		B
**LCSI^50^**	+/Low	+/Low	+/Low	−/Low	+/High		+/High		+/High		+/High		B
**CRRS^51^**				−/Low	−/High						+/High		B
**LCS-ASSS^52^**					+/High					+/High			B
**CLCSS^10^**	+/Moderate	+/Moderate	+/Moderate	−/Moderate	+/High				+/High		+/High		B
**CLCSS-S^53^**				−/Very Low	+/High		+/High						B
**CLCSS-C^54^**	+/Very Low	+/Very Low	+/Very Low	+/High	−/Low		+/High		+/High				B
**CLCSS—SC^55^**				−/Low	+/High						+/High		B
**CLCSS—SM^56^**				+/High	+/High		+/High						B
**IRSS^57^**					+/High								B
**KCSS^58^**			+/Very Low	+/High	−/Low						+/Low		B
**SIS^59^**					+/High								B
**CSS^60^**				+/High	+/High		+/High				+/High		B
**EMIC-PSS^61^**					+/High						+/High		B
**PCRSS^62^**	+/Very Low	+/Very Low	+/Very Low		?						+/High		B
**CRSS^16^**	+/Moderate	+/Moderate	+/Moderate	−/Low	+/High				+/High	+/High	+/High		B
**CIASS^29^**											+/High		B
**SSSQ^63^**	+/Very Low	+/Very Low	+/Moderate	+/High	+/High	+/High					+/High		B
**SSCP^64^**					+/High								B
**PSSS-SS^65^**	+/Moderate	+/Moderate	+/Moderate	+/Moderate	−/Low		+/High			+/High			B
**PSSS-PS^66^**	+/Moderate	+/Moderate	+/Moderate	+/High	+/High	+/High	+/High			+/High	+/High		B
**SRS^67^**											+/High		B
**ISSI^32^**	+/Very Low	+/Very Low	+/Very Low	+/High	+/High					+/High			B
**SRSS II^68^**	+/Low	+/Low	+/Very Low		−/High								B
**SRSS I^69^**	+/Low	+/Low	+/Very Low	+/High	−/High								B
	12			15	25	2	7	0	4	8	15	0	

Symbols indicate the sufficiency of measurement properties: (+) sufficient; (−) insufficient; (±) inconsistent; (?) indeterminate. Shading represents the quality of the evidence, with darker shading indicating higher-quality evidence and blank spaces indicating a lack of evidence.

Abbreviations: CCV, cross-cultural validity; CIASS, Chronic Illness Anticipated Stigma Scale; CL, class; CLCSS, Cataldo Lung Cancer Stigma Scale; CLCSS-C, Chinese version of the Cataldo Lung Cancer Stigma Scale; CLCSS-S, Cataldo Lung Cancer Stigma Scale–Short Version; CLCSS-SC, shortened Chinese version of the Cataldo Lung Cancer Stigma Scale; CLCSS-SM, shortened Mexican version of the Cataldo Lung Cancer Stigma Scale; CRSS, COPD-related Stigma Scale; CrV, criterion validity; CSS, Cancer Stigma Scale; CoV, construct validity; CV, content validity; EMIC-PSS, EMIC Perceived Stigma Subscale; IRSS, Illness-Related Stigma Scale; ISSI, Internalized Stigma of Smoking Inventory; KCSS, Korean Cancer Stigma Scale; ME, measurement error; MI, measurement invariance; PCRSS, Perceived Cancer-Related Stigma Scale; IC, internal consistency; PSSS-PS, Pregnant Smoker Stigma Scale–Public Stigma; PSSS-SS, Pregnant Smoker Stigma Scale–Self-Stigma; REL, reliability; RESP, responsiveness; SRS, Smoking-Related Stigma; SRSS, Smoking-Related Stigma Scale; SSCP, Smoking Stigma During the COVID-19 Pandemic; SSSQ, Smoker Self-Stigma Questionnaire; SV, structural validity.

The highest number of measurement properties (*n = *6) was assessed in the Pregnant Smoker Stigma Scale—Public Stigma^66^ and Lung Cancer Stigma Inventory.^50^ Three scales, namely the Smoker Self-Stigma Questionnaire,^63^ Chinese Version of the Cataldo Lung Cancer Stigma Scale,^54^ and Cataldo Lung Cancer Stigma Scale,^10^ assessed 5 psychometric properties each.

## Discussion

The purpose of this study was to provide practical recommendations for future stigma research, along with evidence-based guidance for researchers and healthcare professionals when selecting, developing, and performing psychometric analysis of stigma scales to assess stigma associated with smoking, COPD, and lung cancer. Our systematic review identified a total of 28 scales that assessed stigma in people who smoke, or those living with COPD or lung cancer.

Our content analysis identified 6 primary themes present in the included scales. The themes we identified were attribution of responsibility and blame, experiences of discrimination, societal labeling and rejection, constrained disclosure, healthcare-associated stigma and consequences of stigma and coping strategies. The emerged themes primarily focused on the drivers, manifestations, and outcomes domains of Stangl’s stigma and discrimination framework, *albeit* with greater detail and additional sub-themes. Existing stigma scales measure stigma either at the individual level (internalized or perceived) or at the public level (public stigma). Meso-level (organizational) and macro-level (policy and social) determinants of stigma were not covered by any scale; future scale development could integrate these important concepts, although these constructs are likely to be more challenging to measure within a scale.

Our audit of question language found widespread use of stigmatizing or potentially stigmatizing terminology. Evidence from qualitative and quantitative studies indicates that people who smoke may experience stigma during smoking-related discussions with their healthcare providers.^68^^,^^71^^,^^72^ It is the ethical responsibility of healthcare providers to avoid using blaming or nihilistic statements in their communication with patients to maintain patient comfort.^72^ Similarly, researchers have a responsibility to ensure that they do not inadvertently instill or reinforce feelings of stigma when measuring stigma in individuals with stigmatized conditions.^73^ Existing stigma scales included labeling, blaming, or oversimplifying language as well as other potentially stigmatizing words such as “disgust.” While measuring stigma without using such stigmatizing terms presents additional challenges, avoidance of these terms or replacement with alternatives and neutral framing should be considered wherever possible for new scales.

In psychometric analyses, we found content validity of the total scale sample was suboptimal and key aspects of content validity were missing to varying extents in the included studies: only 10 studies measured content validity, of which the highest rating was moderate quality of evidence (4 studies). Two studies combined concept elicitation with content validity by involving both experts and target participants, but the remaining studies either conducted concept elicitation or content validity alone, or involved only target participants or experts. As content validity is the most critical measurement property, all 3 aspects of content validity (relevance, comprehensiveness, and comprehensibility) should be explicitly assessed.^38^^,^^74^ Future research should prioritize the optimization of content validity by involving both subject experts and relevant consumers. Moreover, the process of conducting content validation, especially when qualitative methods are used, should be clearly documented. This includes the involvement of skilled interviewers, a description of the interview guide, procedures for recording, transcription, and analysis, as well as the characteristics of the experts or patients involved in the content validity study.

Construct validity consists of 3 parts: structural validity, hypothesis testing, and cross-cultural validity. Fifteen studies assessed structural validity using exploratory factor analysis (EFA to clarify the internal structure), and/or confirmatory factor analysis (CFA to measure the relationship between items and factors in more detail). While COSMIN states that CFA is superior to EFA, most of the studies assessed EFA only. We recommend that future scales use both EFA and CFA. After structural validity, most studies evaluated hypothesis testing for construct validity. Two types of hypothesis testing were commonly used: the hypothesis of a relationship with other scales and the hypothesis of a difference between various subgroups. Three studies tested both types of hypotheses simultaneously, but in most of the studies, hypothesis testing was not explicitly reported. Future studies should clearly report hypothesis testing. Only 2 studies reported cross-cultural validity and measurement invariance, however the evidence was high-quality in both cases.^63^^,^^66^ Measurement invariance is important for ensuring that comparisons, such as across groups, time points or conditions, are meaningful.^75^ Similarly, cross-cultural validity is important to ensure that a scale developed in 1 culture remains valid when adapted to assess stigma in another culture.^76^ Poor measurement invariance and cross-cultural validity can lead to erroneous research conclusions. Therefore, future studies should consider assessing measurement invariance and cross-cultural validity to support generalizability, avoid measurement bias, and promote equity and valid comparison across cultures.

Only 4 studies attempted or claimed to assess criterion validity. Only 1 study used the Cataldo Lung Cancer Stigma Scale (CLCSS), a well-established reference standard for assessing criterion validity.^10^ The remaining studies used either the Rosenberg Self-Esteem Scale^77^ or the Self‑Stigma for Chronic Illnesses scale.^78^ Neither of these 2 scales constitutes a true reference standard; therefore, these analyses should be considered comparisons with other outcome measurement instruments (ie, convergent validity) under hypothesis testing for construct validity, rather than criterion validity.

None of the included scales assessed measurement error or responsiveness. Measurement error indicates the degree to which scale results deviate from the true value. Lower measurement error increases confidence in scores and reduces the number of participants required to detect (small) intervention effects.^79^ Similarly, responsiveness, the detection of the actual change, without over- or under-estimating,^80^ is essential to detect clinically important changes over time in longitudinal studies.^81^ The absence of evidence on these 2 measurement properties undermines the psychometric robustness of the included scales in this review. Future research should rigorously assess these 2 measurement properties of stigma scales in people who smoke and those living with COPD or lung cancer.

In summary, we found no stigma scales that assessed all 9 measurement properties as specified by COSMIN. We identified areas where the assessed measurement properties could be further improved. Although all included stigma scales were rated Class B recommendation, suggesting further research into their measurement properties, the Smoker Self-Stigma Questionnaire,^63^ Internalized Stigma of Smoking Inventory,^32^ and Pregnant Smoker Stigma Scale—Public Stigma^66^ demonstrate comparatively greater potential for use in assessing smoking-related stigma in relevant populations because of their measurement properties. Similarly, the Lung Cancer Stigma Inventory,^50^ the Cataldo Lung Cancer Stigma Scale,^10^ and the Chinese version of the Cataldo Lung Cancer Stigma Scale^54^ could be used to assess stigma among individuals living with lung cancer. Additionally, the COPD-related Stigma Scale may be the most appropriate scale currently for evaluating stigma in patients with COPD.^16^

### Implications for future research and practice

There are increasing calls to address the priority area of stigma associated with lung cancer and COPD, particularly with the rollout of screening programs for lung cancer, where stigma can be a barrier to engagement.^14^^,^^82^ Anti-stigma interventions are mostly in the early stages of evaluation, and accurate assessment of stigma is vital to assess their effectiveness.^14^ The findings of this study offer researchers a foundation upon which to improve existing stigma scales, or to develop new ones that could accurately assess stigma without potentially exacerbating it. The use of person-first and neutral language such as “person who smokes” instead of “smoker,” and “person with lung cancer” instead of “lung cancer patient,” should be promoted in stigma scales. Care should be taken to avoid words such as “disgust” and “unclean.” The IASLC also includes recommended abbreviations where word count limitations exist.

### Strengths and limitations

We used the latest evidence-based resources including IASLC Language Guide 2025, and LCS-CAT 2024, and recently updated COSMIN guidelines to conduct the language audit and review psychometric properties.^39^^,^^40^^,^^83^ We selected COSMIN as the methodological framework for this systematic review because it extends the earlier frameworks of measurement properties of patient-reported outcomes measures. It is widely used and provides a comprehensive, consensus-based set of standards specifically designed to assess the risk of bias in primary studies, evaluate the measurement properties of patient-reported outcome measures, and make recommendations regarding the suitability of existing scales for assessing the construct of interest.^39^ Other available tools, such as the *Quality Criteria for Measurement Properties* proposed by Terwee et al.^84^ and *Evaluating Measures of Patient-Reported Outcomes (EMPRO)* developed by the Spanish Cooperative Investigation Network for Health and Health Service Outcomes Research,^85^ are neither as comprehensive nor as up to date as COSMIN.^86^ For instance, COSMIN separates methodological quality from measurement quality, operationalizes content validity through qualitative inquiry, incorporates modern psychometric approaches (eg, Item Response Theory), and provides guidance for evidence synthesis across studies. These features are either absent or insufficiently developed in the earlier frameworks.^39^ In addition, we reported our results consistent with the PRISMA-COSMIN for Outcome Measurement Instruments.^87^

Despite these strengths, this review is subject to some limitations. First, the study’s inclusion criteria were limited to articles published in English, thereby excluding potentially relevant studies in other languages. Second, the review team opted to follow the rigorous COSMIN guidelines that use highly stringent “worst score counts” approach, meaning that if even 1 item is rated “doubtful” or “inadequate,” higher-quality scores on other items in RoB checklist are disregarded for that measurement property.^74^ Lastly, out of the 28 studies, the second reviewer rated only 10, which may have introduced potential biases or errors. To mitigate this, the reviewer team conducted extensive piloting to increase familiarity with COSMIN and discussed and resolved any uncertainties with the senior authors throughout the process. Additionally, the reviewer team has highly aligned expertise in this area.

## Conclusion

A comprehensively validated stigma scale is needed. Careful consideration should be given to the intentional use of non-stigmatizing, bias-free, and person-first language while developing scales to measure stigma. We recommend the development and use of a non-stigmatizing language compliance checklist for future quantitative studies assessing stigma in people who smoke, or those living with COPD or lung cancer. Assessing the complex experiences of stigma in people living with potentially stigmatizing conditions can be challenging without directly addressing them. Therefore, we suggest presenting item statements of stigma scales in a neutral manner and offering bi-polar response options. This approach avoids introducing stigmatizing language by default. Lastly, more rigorous standards such as COSMIN guidelines and IASLC language guide should be adopted when selecting and developing stigma scales for future research and clinical practice.

## Supplementary Material

kaag026_Supplementary_Data
