# Supplementary material for: COSMIN systematic review of stigma measurement in smoking, COPD, and lung cancer: content analysis, language audit, and psychometrics
Source: Ann Behav Med. 2026 Jun 11;60(1):kaag026. doi: 10.1093/abm/kaag026 (PMC13256004; doi:10.1093/abm/kaag026)
Supplement: kaag026_Supplementary_Data [file kaag026_supplementary_data.docx]

**Table S1**

Search strategies for all databases

| **Database** | **Search Terms** |
| --- | --- |
| **PubMed** | ("Smoking"[Mesh] OR "Tobacco Products"[Mesh] OR "Nicotine"[Mesh] OR "Lung Neoplasms"[Mesh] OR "Pulmonary Disease, Chronic Obstructive"[Mesh] OR "Bronchitis, Chronic"[Mesh] OR "smoking"[tiab] OR "tobacco"[tiab] OR "nicotine"[tiab] OR "cigarette"[tiab] OR "cigarettes"[tiab] OR "lung cancer"[tiab] OR "lung neoplasm"[tiab] OR "lung neoplasms"[tiab] OR "chronic obstructive pulmonary disease"[tiab] OR "COPD"[tiab] OR "respiratory disease"[tiab] OR "respiratory diseases"[tiab] OR "lung disease"[tiab] OR "lung diseases"[tiab] OR "emphysema"[tiab] OR "chronic bronchitis"[tiab])  **AND**  **(**("Social Stigma"[Mesh] OR "stigma"[tiab] OR "stigmas"[tiab] OR "stigmatisation"[tiab] OR "stigmatization"[tiab] OR "stigmatised"[tiab] OR "stigmatized"[tiab]) AND ("Psychometrics"[Mesh] OR "Validation Studies as Topic"[Mesh] OR "Validation Study" [Publication Type] OR "Surveys and Questionnaires"[Mesh] OR "Reproducibility of Results"[Mesh] OR "psychometric"[tiab] OR "psychometrics"[tiab] OR "valid"[tiab] OR "validity"[tiab] OR "validation"[tiab] OR "reliability"[tiab] OR "reliable"[tiab] OR "survey"[tiab] OR "surveys"[tiab] OR "questionnaire"[tiab] OR "questionnaires"[tiab] OR "checklist"[tiab] OR "checklists"[tiab] OR "instrument"[tiab] OR "instruments"[tiab] OR "scale"[tiab] OR "scales"[tiab] OR "instrumentation"[tiab] OR "assessment tool"[tiab] OR "tool"[tiab] OR "tools"[tiab] OR "assessment"[tiab] OR "assessments"[tiab] OR "measurement"[tiab] OR "measurements"[tiab] OR "measure"[tiab] OR "measures"[tiab] OR "self report"[tiab] OR "self reported"[tiab] OR "self-report"[tiab] OR "self-reported"[tiab] OR "index"[tiab] OR "indices"[tiab] OR "inventory"[tiab] OR "inventories"[tiab])**)** |
| **CINAHL Complete (EBSCOhost)** | (MH "Smoking" OR MH "Tobacco Products+" OR MH "Nicotine" OR MH "Lung Neoplasms" OR MH "Pulmonary Disease, Chronic Obstructive+" OR MH "Bronchitis, Chronic" OR TI("smoking" OR "tobacco" OR "nicotine" OR "cigarette" OR "cigarettes" OR "lung cancer" OR "lung neoplasm" OR "lung neoplasms" OR "chronic obstructive pulmonary disease" OR "COPD" OR "respiratory disease" OR "respiratory disease" OR "lung disease" OR "lung diseases" OR "emphysema" OR "chronic bronchitis") OR AB("smoking" OR "tobacco" OR "nicotine" OR "cigarette" OR "cigarettes" OR "lung cancer" OR "lung neoplasm" OR "lung neoplasms" OR "chronic obstructive pulmonary disease" OR "COPD" OR "respiratory disease" OR "respiratory diseases" OR "lung disease" OR "lung diseases" OR "emphysema" OR "chronic bronchitis"))  **AND**  **(**(MH "Stigma" OR TI("stigma" OR "stigmas" OR "stigmatisation" OR "stigmatization" OR "stigmatised" OR "stigmatized") OR AB("stigma" OR "stigmas" OR "stigmatisation" OR "stigmatization" OR "stigmatised" OR "stigmatized")) AND (MH "Psychometrics" OR MH "Validation Studies" OR MH "Surveys+" OR MH "Questionnaires+" OR MH "Reproducibility of Results" OR TI("psychometric" OR "psychometrics" OR "valid" OR "validity" OR "validation" OR "reliability" OR "reliable" OR "survey" OR "surveys" OR "questionnaire" OR "questionnaires" OR "checklist" OR "checklists" OR "instrument" OR "instruments" OR "scale" OR "scales" OR "instrumentation" OR "assessment tool" OR "tool" OR "tools" OR "assessment" OR "assessments" OR "measurement" OR "measurements" OR "measure" OR "measures" OR "self report" OR "self reported" OR "self-report" OR "self-reported" OR "index" OR "indices" OR "inventory" OR "inventories") OR AB("psychometric" OR "psychometrics" OR "valid" OR "validity" OR "validation" OR "reliability" OR "reliable" OR "survey" OR "surveys" OR "questionnaire" OR "questionnaires" OR "checklist" OR "checklists" OR "instrument" OR "instruments" OR "scale" OR "scales" OR "instrumentation" OR "assessment tool" OR "tool" OR "tools" OR "assessment" OR "assessments" OR "measurement" OR "measurements" OR "measure" OR "measures" OR "self report" OR "self reported" OR "self-report" OR "self-reported" OR "index" OR "indices" OR "inventory" OR "inventories"))**)** |
| **Scopus, Advanced Search (Elsevier)** | (TITLE-ABS("smoking" OR "tobacco" OR "nicotine" OR "cigarette" OR "cigarettes" OR "lung cancer" OR "lung neoplasm" OR "lung neoplasms" OR "chronic obstructive pulmonary disease" OR "COPD" OR "respiratory disease" OR "respiratory diseases" OR "lung disease" OR "lung diseases" OR "emphysema" OR "chronic bronchitis"))  **AND**  **(**(TITLE-ABS("stigma" OR "stigmas" OR "stigmatisation" OR "stigmatization" OR "stigmatised" OR "stigmatized")) AND (TITLE-ABS("psychometric" OR "psychometrics" OR "valid" OR "validity" OR "validation" OR "reliability" OR "reliable" OR "survey" OR "surveys" OR "questionnaire" OR "questionnaires" OR "checklist" OR "checklists" OR "instrument" OR "instruments" OR "scale" OR "scales" OR "instrumentation" OR "assessment tool" OR "tool" OR "tools" OR "assessment" OR "assessments" OR "measurement" OR "measurements" OR "measure" OR "measures" OR "self report" OR "self reported" OR "self-report" OR "self-reported" OR "index" OR "indices" OR "inventory" OR "inventories"))**)** |
| **Web of Science, Core Collection All Editions, Advanced Search (Clarivate)** | (TI=("smoking" OR "tobacco" OR "nicotine" OR "cigarette" OR "cigarettes" OR "lung cancer" OR "lung neoplasm" OR "lung neoplasms" OR "chronic obstructive pulmonary disease" OR "COPD" OR "respiratory disease" OR "respiratory disease" OR "lung disease" OR "lung diseases" OR "emphysema" OR "chronic bronchitis") OR AB=("smoking" OR "tobacco" OR "nicotine" OR "cigarette" OR "cigarettes" OR "lung cancer" OR "lung neoplasm" OR "lung neoplasms" OR "chronic obstructive pulmonary disease" OR "COPD" OR "respiratory disease" OR "respiratory diseases" OR "lung disease" OR "lung diseases" OR "emphysema" OR "chronic bronchitis"))  **AND**  **(**(TI=("stigma" OR "stigmas" OR "stigmatisation" OR "stigmatization" OR "stigmatised" OR "stigmatized") OR AB=("stigma" OR "stigmas" OR "stigmatisation" OR "stigmatization" OR "stigmatised" OR "stigmatized")) AND (TI=("psychometric" OR "psychometrics" OR "valid" OR "validity" OR "validation" OR "reliability" OR "reliable" OR "survey" OR "surveys" OR "questionnaire" OR "questionnaires" OR "checklist" OR "checklists" OR "instrument" OR "instruments" OR "scale" OR "scales" OR "instrumentation" OR "assessment tool" OR "tool" OR "tools" OR "assessment" OR "assessments" OR "measurement" OR "measurements" OR "measure" OR "measures" OR "self report" OR "self reported" OR "self-report" OR "self-reported" OR "index" OR "indices" OR "inventory" OR "inventories") OR AB=("psychometric" OR "psychometrics" OR "valid" OR "validity" OR "validation" OR "reliability" OR "reliable" OR "survey" OR "surveys" OR "questionnaire" OR "questionnaires" OR "checklist" OR "checklists" OR "instrument" OR "instruments" OR "scale" OR "scales" OR "instrumentation" OR "assessment tool" OR "tool" OR "tools" OR "assessment" OR "assessments" OR "measurement" OR "measurements" OR "measure" OR "measures" OR "self report" OR "self reported" OR "self-report" OR "self-reported" OR "index" OR "indices" OR "inventory" OR "inventories"))**)** |

**Table S2**

Characteristics of included studies (n=28)

| **Name** | **Country** | **Language** | **Setting** | **Demographic characteristics patients** | | **Disease characteristics** | | **Sample size** |
| --- | --- | --- | --- | --- | --- | --- | --- | --- |
|  |  |  |  | **Mean (SD) age** | **Sex** | **Severity** | **Duration** |  |
| **Stigma Scales for Lung Cancer** |  |  |  |  |  |  |  |  |
| Lung Cancer Stigma and Discrimination Scale [63] | United States | English | Oncology clinics | 64.81 (11.49) | M: 56 (51.9%) F: 52 (48.1%) | Stage I to IV | 9.68 (IQR: 23.46) months | 108 |
| Social Impact Scale [64] | China | Chinese | Tertiary cancer centre | 52.98 (11.08) | M: 174 (61.5%) F: 109 (38.5%) | Stage I to IV | 1 month to >3 years | 313 |
| Social Impact Scale [69] | United States | English | Outpatient chemotherapy visit | 64.04 (8.79) | M: 39 (41.1%) F: 56 (58.9%) | Receiving chemotherapy for stage II–IV | 18.14 (30.35) months | 95 |
| Lung Cancer-Related Stigma Measure [70] | United States | English | Online or face-to-face in clinic | 64.45 (8.69) | M: 24  F: 37 Missing: 1 | Advanced-stage lung cancer (stage III or IV) and had received chemotherapy at least once. | NR | 62 |
| Lung Cancer Stigma Inventory [49] | United States | English | Outpatient oncology clinics | Phase II: 57.05 (6.11) Phase III: 62.80 (10.96) | Phase II:  M: 12 (60%) F: 8 (40%) Phase III:  M: 147 (63.6%) F: 84 (36.4%) | Stage I to IV | NR | Phase II: 20 Phase III: 231 |
| Cancer Responsibility and Regret Scale [57] | United States | English | Medical centre | 64.4 (9.8) | M: 94 (44.1%) F: 119 (55.9%) | Stage I to IV | 65.3 (95.9) weeks | 213 |
| Lung Cancer Stigma Adaptation of the Shame and Stigma Scale [67] | United States | English | Cancer Centre | 70.7 (8.5) | M: 54 (38.3%) F: 87 (61.7%) | Stages IA and IB non-small-cell lung cancer survivors | Time since surgical resection (years): 3.4 (1.2) | 141 |
| Cataldo Lung Cancer Stigma Scale [10] | United States | English | Online survey | 55 (13.7) | M: 42 (28%) F: 109 (72%) | All types of LC and all stages | NR | 186 |
| Short Version of the Cataldo Lung Cancer Stigma Scale [56] | United States | English | Outpatient thoracic and radiation oncology | NR | M: 36 (38%) F: 58 (62%) | Stage I-IV | NR | 94 |
| Chinese Version of the Cataldo Lung Cancer Stigma Scale [51] | China | Chinese | Tertiary hospitals | 58 (3.2) | M: 86 F: 31 | Stage I: 29 Stage II: 51  Stage III: 37 | NR | 150 (117 included) |
| Shortened Version of the Cataldo Lung Cancer Stigma Scale - Chinese version [58] | China | Chinese | Oncology department | 60.56 (10.22) | M: 287 (72.84%) F: 107 (27.16%) | Metastasis: 244 (61.90) | 4.99 (IQR: 11.51) months | 394 |
| Shortened Version of the Cataldo Lung Cancer Stigma Scale - Mexican version [59] | Mexico | Mexican Spanish | NR | 59.19 | M: 162 (61.1%)  F: 103 (38.9%) | Stage I to IV | NR | 265 |
| **Stigma Scales for Cancer, Including Lung Cancer** | | | | | | | | |
| Illness-Related Stigma Scale [65] | United States | English | Two major academic medical centres | 58.34 (9.71) | F: 170 (56.11%) | Stage 0 to IV | 111 (36.6) diagnosed at advanced stage | 303 |
| Korean Cancer Stigma Scale [55] | South Korea | Korean | Inpatient and outpatient | 57.26 (12.25) | F: 60.3% | Stage I to IV | NR | 247 |
| Social Impact Scale [66] | Germany | German | German cancer registries | 60.7 (9.3) | M: 467 (54.4%) F: 391 (45.6%) | Stage I to IV | 1.9 (1.9) years | 858 |
| Cancer Stigma Scale [60] | United Kingdom | English | Online survey | 37.8 years | F: 49% | NA | NA | 1205 |
| Explanatory Model Interview Catalogue (EMIC) - Perceived Stigma Subscale [68] | Canada | English | Oncology clinics | 62.95 (11.58) | M: 44.2% F: 55.8% | Local: 75.5 Advanced: 24.5 | 1.29 (0.85) years | 206 (lung cancer: 107) |
| Perceived Cancer-Related Stigma Scale [54] | United States | English | Participants were contacted by telephone or mail. | 66.9 (11.18) | M: 95 (55.2%) F: 77 (44.8%) | Stage IV | NR | 172 |
| **Stigma Scales for COPD** | | | | | | | | |
| COPD-related Stigma Scale [16] | United States | English | Pulmonary and medical clinics (mailed survey) | 64.06 (7.27) | M: 75 (50.7%) F: 73 (49.3%) | FEV1/FVC (%): 62.1 (15.3) | 8.84 (7.87) years | 148 |
| Chronic Illness Anticipated Stigma Scale (CIASS) [29]  (Sub-scale) | Australia | English | Online | NR (overall) | NR (overall) | NR | NR | 556 |
| **Smoking-Related Stigma Scales** |  |  |  |  |  |  |  |  |
| Smoker Self-Stigma Questionnaire [46] | United States | English | Online survey | 37.1 (10.7) | M: 51.4% F: 48.1% | NA | NA | 592 |
| Smoking Stigma During the COVID-19 Pandemic (SS-CP) [62] | South Korea | Korean | Online | NR | NR | NR | NR | 7293 |
| Pregnant Smoker Stigma Scale - Self-Stigma [48] | France | French | Maternity hospital | 29.07 (5.62) | F: 100% | NA | Time before delivery: 103.38 (58.33) days | 142 |
| Pregnant Smoker Stigma Scale – Public Stigma [47] | France | French | Online | 33.95 (13.95) | M: 110 (32.2%)  F: 229 (67%) | NA | NA | 342 |
| Smoking-Related Stigma (Three-Item Measure) [71] | Mexico, Uruguay | Spanish | Household survey | Mean age ranged 39 and 43 years | M (Mexican): 62–63% Uruguay: Proportion equally distributed. | NA | NA | Mexico (n = 6670) Uruguay (n = 3296) |
| Internalized Stigma of Smoking Inventory [50] | United States | English | Inpatient psychiatry ward | NR | M: 490 51.3% F: 466 48.7% | NA | NA | 956 |
| Smoking-Related Stigma Scale [53] | United States | English | Telephone survey | 43.7 (15.1) | F: 372 (43.7%) | NA | NA | 811 |
| Smoker-Related Stigma Scale [52] | United States | English | Telephone survey | NR | F: 366 (43 %) | NA | NA | 816 |

NA: Not applicable, NR: Not reported

**Table S3**

Overview of item development and content validity of stigma scales (n=28)

| **Reference** | **Intended context of use** | **Item development process** | **Cognitive Interviewing/ Consumers’ involvement** |
| --- | --- | --- | --- |
| **Stigma Scales for Lung Cancer** | | | |
| Lung Cancer Stigma and Discrimination Scale [63] | To investigate whether three facets of lung cancer stigma (internalized stigma, constrained disclosure, and perceived subtle discrimination) uniquely predicted psychological and physical health-related adjustment to lung cancer across 12 weeks. | - Internalized stigma and constrained disclosure subscales were adapted from CRRS and HIV Stigma Scale. - Perceived subtle discrimination subscale (50 items) was constructed de novo using existing HIV and racial stigma scales. | NR |
| Social Impact Scale [64] | To examine the level of stigma and identify the correlates of stigma among people with lung cancer. | - The 24-item Chinese version of SIS was used to assess stigma. | NR |
| Social Impact Scale [69] | To assess the relationship between perceived stigma and depressive symptomatology in people with lung cancer. | - The original 24-item SIS was used to evaluate the perceived social impact of lung cancer. | NR |
| Lung Cancer-Related Stigma Measure [70] | To examine the influence of stigma on social support, social constraints, symptom severity, symptom interference, and quality of life in people with advanced lung cancer. | - Adapted from the validated stigma items for cancer patients. | NR |
| Lung Cancer Stigma Inventory [49] | To assess the lung cancer stigma among people with lung cancer using a psychometrically robust scale | - Building on previously reported concept elicitation (Phase I) work, Phase II of LCSI development involved item generation and refinement, informed by literature review, provider input, and patient (N=20) feedback. - Phase III focused on initial psychometric analysis. | - One of three lung cancer expert teams (each including a thoracic oncology clinician and a psychosocial clinician) rated each for relevance and clarity. - A hybrid approach was used which combined “think-aloud”, “post-test” debriefing, and referrals to further resources. - Interviewees were asked to comment on the scale instructions and the response options. |
| Cancer Responsibility and Regret Scale [57] | To evaluate rates and intensity of these types of experiences and to characterize the extent to which they are linked with smoking status and psychological adjustment in those living with lung cancer. | - An initial pool of 23 items was developed from existing stigma-related scales and qualitative input. - Scale was reduced to 11 items by factor analysis. | - Feedback was incorporated from support group members and healthcare professionals during the item refinement procedure. |
| Lung Cancer Stigma Adaptation of the Shame and Stigma Scale [67] | To study the relationship between stigma, posttraumatic growth, and psychological distress. | - Adapted the original Shame and Stigma Scale. Subscales specific to head and neck cancers (e.g., shame with appearance) were removed, and items for guilt and regret related to smoking behaviour were added. | NR |
| Cataldo Lung Cancer Stigma Scale [10] | To measure health-related stigma in people with lung cancer. | - Seven experts were asked to review the HIV Perceived Stigma Scale and assess clarity, relevance and comprehensiveness of items in relation to stigma in lung cancer. - Based on this review and additional items, a 46-item scale was proposed for further analysis. | - Reviewed by experts in psychology, oncology, and sociology for item clarity and relevance. |
| Short Version of the Cataldo Lung Cancer Stigma Scale [56] | To reduce survey burden while maintaining psychometric reliability. | - Items reduced from 31 to 21 using EFA to identify item redundancy and refine factors. | - In the parent study, input was gathered from oncology experts and patients in earlier stages of CLCSS development. - Self-report, written surveys were administered by the researcher in person to people with lung cancer, which was followed by a semi-structured interview. |
| Chinese Version of the Cataldo Lung Cancer Stigma Scale [51] | To validate and culturally adapt the CLCSS for Chinese lung cancer patients. | - The original 31-item CLCSS was translated and culturally adapted to the Chinese population. - Six culturally irrelevant items were removed. Based on the response to open-ended question by 30 people with lung cancer and the in-depth interview by 6 people with lung cancer, 13 new items were added. - The final version consisted of 38 items, reduced to 27 items across four subscales by EFA. The scale is pilot tested among 20 people with lung cancer. | - Feedback from oncology experts and Chinese patients ensured clarity and cultural alignment. - Open-ended surveys and interviews informed additional items and revisions. |
| Shortened Version of the Cataldo Lung Cancer Stigma Scale - Chinese version [58] | To investigate a predictive model of psychosocial supportive care among Chinese people with lung cancer. | - Items from the 31-item CLCSS were analysed using EFA and CFA. - Final 22 items were validated across four subscales. | NR |
| Shortened Version of the Cataldo Lung Cancer Stigma Scale - Mexican version [59] | To determine the psychometric properties of the CLCSS in a Mexican sample of  lung cancer patients. | - Adapted and translated from Cataldo Lung Cancer Stigma Scale (CLCSS) | - Pilot testing in 15 patients living with lung cancer. - Content validation by a group of expert psychologists, pulmonologists, and oncologists |
| **Stigma Scales for Cancer, Including Lung Cancer** | | | |
| Illness-Related Stigma Scale [65] | To assess the impact of illness-related stigma on patient-provider interactions and receipt of smoking cessation treatment. | - Developed with 5 items addressing dimensions of internalized shame and fear of disclosure. | NR |
| Korean Cancer Stigma Scale [55] | To assess health-related stigma across cancers. | - Developed using translation, cultural adaptation, and iterative refinement of the CLCSS. - Translation (2 bilingual nursing Professors) and back-translation (an English expert) processes were conducted. - Items were revised for cultural relevance and tested with a pilot group of 15 patients. - EFA and CFA validated the 24-item scale. | - Feedback integrated from a panel of three nursing professors, one oncology nurse, and 15 patients with cancer to test feasibility and content validity. |
| Social Impact Scale [66] | To assess the levels of stigmatization and its impact on quality of life among a large sample including four major tumour entities. | - The 24-item German version of SIS was used to assess stigma. | NR |
| Cancer Stigma Scale [60] | To assess public stigma toward cancer in non-patient populations. | - Initial pool of 481 items reduced to 25 after exploratory and confirmatory factor analyses. | - Feedback incorporated from cancer researchers and public surveys. - 57 students were involved to assess the clarity of the first item set. |
| Explanatory Model Interview Catalogue (EMIC) - Perceived Stigma Subscale [68] | To study the psychosocial impact of stigma and its effects on positive health changes (PHCs) in head and neck and lung cancer. | - Original 13-item EMIC stigma subscale adapted for cancer settings. - Items were rephrased for lung cancer and its treatment. | NR |
| Perceived Cancer-Related Stigma Scale [54] | To evaluate stigma related to cancer and its effects on psychological distress and care. | - Developed with 6 investigator-designed items addressing guilt, shame, and embarrassment about cancer. - Items were rated on a 5-point Likert scale. Items underwent thematic analysis but were not previously validated. | - Input from a lung cancer support group informed item generation. - Feedback integrated into item clarity but not formally validated for other cancer groups. |
| **Stigma Scales for COPD** | | | |
| COPD-related Stigma Scale [16] | To understand the underlying stigma processes in people with COPD. | - Developed using a preliminary 60-item scale derived from qualitative studies on COPD stigma and the HIV Stigma Scale. - Reviewed by experts, refined through cognitive interviews to improve clarity and accuracy, resulting in a 51-item version. - The 24-item final scale was derived by EFA. | - Reviewed by experts to ensure clarity and relevance. - Nineteen people with COPD (6 men and 13 women) participated in semi-structured cognitive interviews. |
| Chronic Illness Anticipated Stigma Scale (CIASS) [29]  (Sub-scale) | To compare levels of ‘anticipated’ stigma and experience of care in general practice between people who currently smoke and previously smoked, living with COPD, other chronic illnesses, or those with no chronic conditions. | - The healthcare worker subscale of CIASS included 5 items specifically addressing stigma expectations in medical settings. | NR |
| **Smoking-Related Stigma Scales** | | | |
| Smoker Self-Stigma Questionnaire [46] | To assess the extent to which smoking stigma is a barrier to successful outcomes from smoking cessation attempts. | - Items were selected from 13 validated addiction and behavioural stigma scales, producing 223 items. - Three experts grouped items by semantic similarity and divided them into the stigma domains - Additional items reflecting smokers’ experiences from qualitative studies were added, resulting in an 88-item pool. - Items were rated using a 4-point Likert scale for face validity by eight experts. - 45 highest-quality items were retained. | - Eight experts assessed each item's face validity, assigned it to self-stigma facets (enacted, felt, internalized). - Perspectives from people who smoke, as captured in qualitative research, were also incorporated. |
| Smoking Stigma During the COVID-19 Pandemic (SS-CP) [62] | To examine the change in social and personal stigmas after the COVID-19 pandemic. | - Items developed from previous stigma scales for people who smoke and don't smoke. | NR |
| Pregnant Smoker Stigma Scale - Self-Stigma [48] | To understand how self-stigma might affect mental health and smoking cessation in pregnant women who smoke. | - Developed based on the Public Stigma Scale (P3S-PS). - Items were adapted to reflect self-stigma by three psychologists and two psychiatrists. | - The item adaptation was performed by members of the research team (three health psychologists and two psychiatrists). |
| Pregnant Smoker Stigma Scale – Public Stigma [47] | To assess the public stigma surrounding pregnant women who smoke. | - Items were generated from qualitative analysis of 100 participants assessing stigma-related cognitions, emotions, and behaviours. - Initial item pool (n = 32) reviewed by 21 experts in perinatal care and addiction treatment. - EFA conducted on a final 26-item scale with validation across four dimensions. | - Feedback obtained from 21 experts (psychiatrists, psychologists, midwives, and nurses) on clarity and relevance. - Items were tested in general population (n = 30) tested for item clarity. |
| Smoking-Related Stigma (Three-Item Measure) [71] | To understand the role of social norms and socioeconomic status in smoking-related stigma. | - Three items were designed to capture emotional discomfort, negative stereotypes, and perceived marginalization. - Items were adapted from smoking-related stigma scales and were modified for Latin American contexts. | NR |
| Internalized Stigma of Smoking Inventory [50] | To assess self-stigma, felt stigma, and enacted stigma among people who smoke. | - Developed by modifying three ISMI subscales (Stereotype Endorsement, Social Withdrawal, Perceived Discrimination). - A team of experts adapted the items. - EFA and CFA reduced the initial item pool to an 8-item scale validated across three subscales. | - Expert feedback from specialists in smoking cessation, substance treatment, and mental illness was incorporated. |
| Smoking-Related Stigma Scale [53] | To study the prevalence and consequences of smoking stigma. | - Developed with 5 items adapted from the Devaluation-Discrimination Scale by Link et al. (1989). - Focused on dimensions of perceived devaluation, differential treatment, and behavioural consequences. | - Scale was pretested in 20 participants |
| Smoker-Related Stigma Scale [52] | To examine the role of attribution, fear, tobacco control policies, power and social norms in the formation of smoking-related stigma. | - Developed from a commonly used mental health stigma scale (12 items). - Modified for smoker-related stigma. Final measure reduced to five items after pilot testing in 20 participants. | - Scale development study involving 20 participants. |

NR: Not reported

**Table S4**

Results on the risk of bias (RoB), raw results, and ratings for each study on a measurement property; and the summarized result, overall rating and level of evidence for each stigma scale

| No | Scale | Result | Rating | Quality of study | Sample size | Quality of Evidence |
| --- | --- | --- | --- | --- | --- | --- |
| 1 | **Lung Cancer Stigma and Discrimination Scale (Williamson et al., 2022)** | |  |  |  |  |
|  | Structural validity |  |  |  |  |  |
|  | Internal consistency | Internalized stigma: 0.75 Constrained disclosure (Spearman-Brown coefficient): 0.74 Perceived subtle experiences of discrimination: 0.95 | + | VG | 108 | High |
|  | Cross-cultural validity |  |  |  |  |  |
|  | Reliability |  |  |  |  |  |
|  | Measurement error |  |  |  |  |  |
|  | Criterion validity |  |  |  |  |  |
|  | Construct validity | Convergent validity | + | VG | 108 | High |
|  | Responsiveness |  |  |  |  |  |
| 2 | **Social Impact Scale (Liu et al., 2020)** | |  |  |  |  |
|  | Structural validity |  |  |  |  |  |
|  | Internal consistency | 0.917 (Overall scale) | ? |  |  |  |
|  | Cross-cultural validity |  |  |  |  |  |
|  | Reliability |  |  |  |  |  |
|  | Measurement error |  |  |  |  |  |
|  | Criterion validity |  |  |  |  |  |
|  | Construct validity | Convergent and known groups validity | + | VG | 283 | High |
|  | Responsiveness |  |  |  |  |  |
| 3 | **Social Impact Scale (Gonzalez & Jacobsen, 2012)** | |  |  |  |  |
|  | Structural validity |  |  |  |  |  |
|  | Internal consistency | >0.86 | + | VG | 95 | Moderate |
|  | Cross-cultural validity |  |  |  |  |  |
|  | Reliability |  |  |  |  |  |
|  | Measurement error |  |  |  |  |  |
|  | Criterion validity |  |  |  |  |  |
|  | Construct validity | Stigma and depression (Sample size) | + | VG | 95 | Moderate |
|  | Responsiveness |  |  |  |  |  |
| 4 | **Lung Cancer-Related Stigma Measure (Johnson et al., 2019)** | |  |  |  |  |
|  | Structural validity |  |  |  |  |  |
|  | Internal consistency |  |  |  |  |  |
|  | Cross-cultural validity |  |  |  |  |  |
|  | Reliability |  |  |  |  |  |
|  | Measurement error |  |  |  |  |  |
|  | Criterion validity |  |  |  |  |  |
|  | Construct validity | KGV (Sample size) | + | VG | 62 | Low |
|  | Responsiveness |  |  |  |  |  |
| 5 | **Lung Cancer Stigma Inventory (Hamann et al., 2018, Ostroff et al., 2018)** | |  |  |  |  |
|  | Structural validity | 3 factors explained 43% of variance | - | D | 195 | Low |
|  | Internal consistency | 0.74-0.90 | + | VG | 195 | High |
|  | Cross-cultural validity |  |  |  |  |  |
|  | Reliability | High test–retest correlation (r =0.91) within 60 days | + | VG | 55 | High |
|  | Measurement error |  |  |  |  |  |
|  | Criterion validity | CLCSS and LCSI | + | VG | 195 | High |
|  | Construct validity | Lung cancer stigma was moderately correlated with depressive mood (r = 0.44) | + | VG | 231 | High |
|  | Responsiveness |  |  |  |  |  |
| 6 | **Cancer Responsibility and Regret Scale (Criswell et al., 2016)** | |  |  |  |  |
|  | Structural validity | <50% variance explained | - | A | 213 | Low |
|  | Internal consistency | 0.64-0.84 | - | VG | 213 | High |
|  | Cross-cultural validity |  |  |  |  |  |
|  | Reliability |  |  |  |  |  |
|  | Measurement error |  |  |  |  |  |
|  | Criterion validity |  |  |  |  |  |
|  | Construct validity | Greater stigma in people who smoke than who never smoke | + | VG | 213 | High |
|  | Responsiveness |  |  |  |  |  |
| 7 | **Lung Cancer Stigma Adaptation of the Shame and Stigma Scale (Shen et al., 2015)** | |  |  |  |  |
|  | Structural validity |  |  |  |  |  |
|  | Internal consistency | 0.77-0.79 | + | VG | 141 | High |
|  | Cross-cultural validity |  |  |  |  |  |
|  | Reliability |  |  |  |  |  |
|  | Measurement error |  |  |  |  |  |
|  | Criterion validity |  |  |  |  |  |
|  | Construct validity | Positive association between stigma and distress. | + | VG | 141 | High |
|  | Responsiveness |  |  |  |  |  |
| 8 | **Cataldo Lung Cancer Stigma Scale (Cataldo et al., 2011)** | |  |  |  |  |
|  | Structural validity | 4-factor solution explained 57% variance  Factor loading 0.35 and above  >10% items have factor loading >0.30 | - | A | 186 | Moderate |
|  | Internal consistency | 0.75-0.97 | + | VG | 186 | High |
|  | Cross-cultural validity |  |  |  |  |  |
|  | Reliability |  |  |  |  |  |
|  | Measurement error |  |  |  |  |  |
|  | Criterion validity | RSES (-0.723) | + | VG | 186 | High |
|  | Construct validity | Stigma and QoL (-0.618) | + | VG | 186 | High |
|  | Responsiveness |  |  |  |  |  |
| 9 | **Short Version of the Cataldo Lung Cancer Stigma Scale (Carter-Harris & Hall, 2014)** | |  |  |  |  |
|  | Structural validity | 68% variance explained, factor loading >0.3, >10% multiple loading | - | D | 94 | Very Low |
|  | Internal consistency | 0.89-0.92 | + | VG | 94 | High |
|  | Cross-cultural validity |  |  |  |  |  |
|  | Reliability | Correlations between time 1 and time 2 scores were significant for all the factors  (r = .72-.82, all p’s < .001). | + | VG | 94 | High |
|  | Measurement error |  |  |  |  |  |
|  | Criterion validity |  |  |  |  |  |
|  | Construct validity |  |  |  |  |  |
|  | Responsiveness |  |  |  |  |  |
| 10 | **Chinese Version of the Cataldo Lung Cancer Stigma Scale (Yang et al., 2014)** | |  |  |  |  |
|  | Structural validity | Four factors explained 58.6% of the total variance in the results, factor loading of > 0.4 | + | VG | 117 | High |
|  | Internal consistency | 0.599 to 0.868 | - | VG | 117 | Low |
|  | Cross-cultural validity |  |  |  |  |  |
|  | Reliability | 0.633 to 0.881 | + | VG | 117 | High |
|  | Measurement error |  |  |  |  |  |
|  | Criterion validity | Rosenberg Self-esteem Scale | + | VG | 117 | High |
|  |  | Self-Rating Depression Scale |  |  |  |  |
|  | Construct validity |  |  |  |  |  |
|  | Responsiveness |  |  |  |  |  |
| 11 | **Shortened Version of the Cataldo Lung Cancer Stigma Scale - Chinese version (Lv et al., 2022)** | |  |  |  |  |
|  | Structural validity | Final model explained 64.4% of the total variance  >10% multiple factors loading | - | VG | 204 | Low |
|  | Internal consistency | >0.771 | + | VG | 394 | High |
|  | Cross-cultural validity |  |  |  |  |  |
|  | Reliability |  |  |  |  |  |
|  | Measurement error |  |  |  |  |  |
|  | Criterion validity |  |  |  |  |  |
|  | Construct validity | Stigma education and employment level (significant association) | + | VG | 394 | High |
|  | Responsiveness |  |  |  |  |  |
| 12 | **Shortened Version of the Cataldo Lung Cancer Stigma Scale - Mexican version (Flores-Juárez et al., 2024)** | |  |  |  |  |
|  | Structural validity | NFI = 0.916; TLI = 0.950; **CFI = 0.959**; AGFI = 0.887; SRMR = 0.0673 and RMSEA = 0.057 | + | VG | 265 | High |
|  | Internal consistency | Scale: 0.85 | + | VG | 265 | High |
|  |  | Sub-scales: 0.75-0.88 |  |  |  |  |
|  | Cross-cultural validity |  |  |  |  |  |
|  | Reliability | Test-retest reliability: 0.85 | + | VG | 265 | High |
|  | Measurement error |  |  |  |  |  |
|  | Criterion validity |  |  |  |  |  |
|  | Construct validity |  |  |  |  |  |
|  | Responsiveness |  |  |  |  |  |
| 13 | **Illness-Related Stigma Scale (Neil et al., 2020)** | |  |  |  |  |
|  | Structural validity |  |  |  |  |  |
|  | Internal consistency | 0.76 | + | VG | 303 | High |
|  | Cross-cultural validity |  |  |  |  |  |
|  | Reliability |  |  |  |  |  |
|  | Measurement error |  |  |  |  |  |
|  | Criterion validity |  |  |  |  |  |
|  | Construct validity |  |  |  |  |  |
|  | Responsiveness |  |  |  |  |  |
| 14 | **Korean Cancer Stigma Scale (So et al., 2017)** | |  |  |  |  |
|  | Structural validity | Eigen values>1, variances explained: 65.7, factor loading >0.30 | + | VG | 247 | High |
|  | Internal consistency | 0.62-0.86 | - | VG | 247 | Low |
|  | Cross-cultural validity |  |  |  |  |  |
|  | Reliability |  |  |  |  |  |
|  | Measurement error |  |  |  |  |  |
|  | Criterion validity |  |  |  |  |  |
|  | Construct validity | Distress group had a significantly higher perceived cancer stigma than the non-distress group (insomnia, anxiety, and depression domains): Indirectness (scores were not only from lung cancer) | + | VG | 247 | Low |
|  | Responsiveness |  |  |  |  |  |
| 15 | **Social Impact Scale (Ernst et al., 2017)** | |  |  |  |  |
|  | Structural validity |  |  |  |  |  |
|  | Internal consistency | 0.81-0.89 | + | VG | 858 | High |
|  | Cross-cultural validity |  |  |  |  |  |
|  | Reliability |  |  |  |  |  |
|  | Measurement error |  |  |  |  |  |
|  | Criterion validity |  |  |  |  |  |
|  | Construct validity |  |  |  |  |  |
|  | Responsiveness |  |  |  |  |  |
| 16 | **Cancer Stigma Scale (Marlow et al., 2015)** | |  |  |  |  |
|  | Structural validity | CFI = 0.942, TLI = 0.933, SRMR = 0.064, RMSEA = 0.052. | + | VG | 238 | High |
|  | Internal consistency | 0.73-0.87 | + | VG | 462 | High |
|  | Cross-cultural validity |  |  |  |  |  |
|  | Reliability | Correlations between time 1 and time 2 scores were significant for all the factors (r = .72-.82, all p’s < .001). | + | VG | 249 | High |
|  | Measurement error |  |  |  |  |  |
|  | Criterion validity |  |  |  |  |  |
|  | Construct validity | Differences in mean scores across the five cancer types | + | VG | 1205 | High |
|  | Responsiveness |  |  |  |  |  |
| 17 | **Explanatory Model Interview Catalogue (EMIC) - Perceived Stigma Subscale (Lebel et al., 2013)** | |  |  |  |  |
|  | Structural validity |  |  |  |  |  |
|  | Internal consistency | 0.82 | + | VG | 206 | High |
|  | Cross-cultural validity |  |  |  |  |  |
|  | Reliability |  |  |  |  |  |
|  | Measurement error |  |  |  |  |  |
|  | Criterion validity |  |  |  |  |  |
|  | Construct validity | Lung cancer attracts greater stigma than head and neck cancer | + | VG | 206 | High |
|  | Responsiveness |  |  |  |  |  |
| 18 | **Perceived Cancer-Related Stigma Scale (LoConte et al., 2008)** | |  |  |  |  |
|  | Structural validity |  |  |  |  |  |
|  | Internal consistency | 0.75  Not enough information on unidimensionality | ? |  |  |  |
|  | Cross-cultural validity |  |  |  |  |  |
|  | Reliability |  |  |  |  |  |
|  | Measurement error |  |  |  |  |  |
|  | Criterion validity |  |  |  |  |  |
|  | Construct validity |  | + | VG | 172 | High |
|  | Responsiveness |  |  |  |  |  |
| 19 | **COPD-related Stigma Scale**  **(Woo et al., 2023)** | |  |  |  |  |
|  | Structural validity | 4-factor solution explained 65.15% of the total variance  >10% items have factor loading on multiple factors | - | A | 116 | Low |
|  | Internal consistency | Scale: 0.93  Sub-scales: 0.80-0.95 | + | VG | 148 | High |
|  | Cross-cultural validity |  |  |  |  |  |
|  | Reliability |  |  |  |  |  |
|  | Measurement error |  |  |  |  |  |
|  | Criterion validity |  | + | VG | 148 | High |
|  | Construct validity |  | + | VG | 148 | High |
|  | Responsiveness |  |  |  |  |  |
| 20 | **Chronic Illness Anticipated Stigma Scale (CIASS)**  **(Madawala et al., 2023) (Sub-scale)** | |  |  |  |  |
|  | Structural validity |  |  |  |  |  |
|  | Internal consistency |  |  |  |  |  |
|  | Cross-cultural validity |  |  |  |  |  |
|  | Reliability |  |  |  |  |  |
|  | Measurement error |  |  |  |  |  |
|  | Criterion validity |  |  |  |  |  |
|  | Construct validity |  | + | VG | 556 | High |
|  | Responsiveness |  |  |  |  |  |
| 21 | **Smoker Self-Stigma Questionnaire (Geist et al., 2023)** | |  |  |  |  |
|  | Structural validity | CFI=0.961, TLI=0.954, RMSEA=0.057, SRMR=0.037 | + | A | 296 | High |
|  | Internal consistency | Scale: 0.95  Enacted: 0.85- 0.90 | + | VG | 296 | High |
|  | Cross-cultural validity | No important difference found between two samples | + | VG | 296 | High |
|  | Reliability |  |  |  |  |  |
|  | Measurement error |  |  |  |  |  |
|  | Criterion validity |  |  |  |  |  |
|  | Construct validity | Hispanic any race: 93.65 (20.35)  Non-Hispanic White: 93.76 (22.71)  Non-Hispanic Black: 73.55 (24.20) | + | VG | 558 | High |
|  | Responsiveness |  |  |  |  |  |
| 22 | **Smoking Stigma During the COVID-19 Pandemic (SS-CP)**  **(Jung & Lee, 2023)** | |  |  |  |  |
|  | Structural validity |  |  |  |  |  |
|  | Internal consistency | 0.83-0.86 | + | VG | 7293 | High |
|  | Cross-cultural validity |  |  |  |  |  |
|  | Reliability |  |  |  |  |  |
|  | Measurement error |  |  |  |  |  |
|  | Criterion validity |  |  |  |  |  |
|  | Construct validity |  |  |  |  |  |
|  | Responsiveness |  |  |  |  |  |
| 23 | **Pregnant Smoker Stigma Scale - Self-Stigma**  **(Loyal et al., 2023)** | |  |  |  |  |
|  | Structural validity | CFI: 0.981-0.986 | + | A | 142 | Moderate |
|  | Internal consistency | 0.61-0.91 | - | VG | 142 | Low |
|  | Cross-cultural validity |  |  |  |  |  |
|  | Reliability | ICC: >0.75 | + | VG | 142 | High |
|  | Measurement error |  |  |  |  |  |
|  | Criterion validity |  |  |  |  |  |
|  | Construct validity |  | + | VG | 142 | High |
|  | Responsiveness |  |  |  |  |  |
| 24 | **Pregnant Smoker Stigma Scale – Public Stigma (Loyal et al., 2022)** | |  |  |  |  |
|  | Structural validity | 58% variance explained, loading >30, <10% multiple loading | + | A | 342 | High |
|  | Internal consistency | 0.77-0.93 | + | VG | 342 | High |
|  | Cross-cultural validity | The configural model was satisfactory for gender and smoking status | + | VG | 342 | High |
|  | Reliability | ICC>.75 | + | VG | 342 | High |
|  | Measurement error |  |  |  |  |  |
|  | Criterion validity |  |  |  |  |  |
|  | Construct validity |  | + | VG | 342 | High |
|  | Responsiveness |  |  |  |  |  |
| 25 | **Smoking-Related Stigma (Three-Item Measure), (Lozano et al., 2019)** | |  |  |  |  |
|  | Structural validity |  |  |  |  |  |
|  | Internal consistency |  |  |  |  |  |
|  | Cross-cultural validity |  |  |  |  |  |
|  | Reliability |  |  |  |  |  |
|  | Measurement error |  |  |  |  |  |
|  | Criterion validity |  |  |  |  |  |
|  | Construct validity |  | + | VG | Mexico (n = 6670)  Uruguay (n = 3296) | High |
|  | Responsiveness |  |  |  |  |  |
| 26 | **Internalized Stigma of Smoking Inventory (Brown-Johnson et al., 2015)** | |  |  |  |  |
|  | Structural validity | SRMR=0.04 | + | VG | 478 | High |
|  | Internal consistency | 0.70-0.81 | + | VG | 956 | High |
|  | Cross-cultural validity |  |  |  |  |  |
|  | Reliability |  |  |  |  |  |
|  | Measurement error |  |  |  |  |  |
|  | Criterion validity |  |  |  |  |  |
|  | Construct validity | GLMs explain 21–30%; expected directions with readiness to quit, other stigma, dependence | + | VG | 956 | High |
|  | Responsiveness |  |  |  |  |  |
| 27 | **Smoking-Related Stigma Scale (Stuber et al., 2009)** | |  |  |  |  |
|  | Structural validity |  |  |  |  |  |
|  | Internal consistency | 0.65-0.7 | - | VG | 816 | High |
|  | Cross-cultural validity |  |  |  |  |  |
|  | Reliability |  |  |  |  |  |
|  | Measurement error |  |  |  |  |  |
|  | Criterion validity |  |  |  |  |  |
|  | Construct validity |  |  |  |  |  |
|  | Responsiveness |  |  |  |  |  |
| 28 | **Smoker-Related Stigma Scale**  **(Stuber et al., 2008)** | |  |  |  |  |
|  | Structural validity | 4/5 items loaded on one factor  No explanation of >50% variance explained, Kaiser criteria Factor loadings of 0.32 or greater | + | VG | 816 | High |
|  | Internal consistency | 0.61 | - | VG | 816 | High |
|  | Cross-cultural validity |  |  |  |  |  |
|  | Reliability |  |  |  |  |  |
|  | Measurement error |  |  |  |  |  |
|  | Criterion validity |  |  |  |  |  |
|  | Construct validity |  |  |  |  |  |
|  | Responsiveness |  |  |  |  |  |

+ sufficient; – insufficient; ± inconsistent; ? indeterminate

Abbreviations: CFI: Comparative Fit Index; CI: Comparisons between instruments; CTT: Classical Test Theory; CV: Cross-Cultural Validity; DIF: Differential Item Functioning; ICC: Intraclass Correlation Coefficient; IRT: Item Response Theory; KG: Known Groups; MI: Measurement Invariance; N: Number; NA: Not Assessed; r: Correlation; Resid corr: Residual Correlation; RMSEA: Root Mean Square Error of Approximation; RoB: Risk of Bias; SRMR: Standardized Root Mean Square Residual; TLI: Tucker-Lewis Index.
